# Chromosome 1q21.2 and additional loci influence risk of spontaneous coronary artery dissection and myocardial infarction

**DOI:** 10.1038/s41467-020-17558-x

**Published:** 2020-09-04

**Authors:** Jacqueline Saw, Min-Lee Yang, Mark Trinder, Catherine Tcheandjieu, Chang Xu, Andrew Starovoytov, Isabelle Birt, Michael R. Mathis, Kristina L. Hunker, Ellen M. Schmidt, Linda Jackson, Natalia Fendrikova-Mahlay, Matthew Zawistowski, Chad M. Brummett, Sebastian Zoellner, Alexander Katz, Dawn M. Coleman, Kirby Swan, Christopher J. O’Donnell, Million Veteran Program, Themistocles L. Assimes, Christopher J. O’Donnell, Xiang Zhou, Jun Z. Li, Heather L. Gornik, Themistocles L. Assimes, James C. Stanley, Liam R. Brunham, Santhi K. Ganesh

**Affiliations:** 1 Vancouver General Hospital, Division of Cardiology, University of British Columbia, Vancouver, BC Canada; 2 Division of Cardiovascular Medicine, Department of Internal Medicine, University of Michigan Medical School, Ann Arbor, MI USA; 3 Department of Human Genetics, University of Michigan Medical School, Ann Arbor, MI USA; 4 Centre for Heart Lung Innovation, Department of Medicine, University of British Columbia, Vancouver, BC Canada; 5 VA Palo Alto Health Care System, Palo Alto, CA USA; 6 Division of Cardiovascular Medicine, Department of Medicine, Stanford University School of Medicine, Stanford, CA USA; 7 Department of Pediatrics, Division of Pediatric Cardiology, Stanford University School of Medicine, Stanford, CA USA; 8 Department of Biostatistics and Center for Statistical Genetics, University of Michigan School of Public Health, Ann Arbor, MI USA; 9 Department of Anesthesiology, Michigan Medicine, University of Michigan, Ann Arbor, MI USA; 10 Heart and Vascular Institute, Cleveland Clinic, Cleveland, OH 44195 USA; 11 Medical Genomics and Metabolic Genetics Branch, National Human Genome Research Institute, National Institutes of Health, Bethesda, MD 20892 USA; 12 Vascular Surgery Section, Department of Surgery, Michigan Medicine, University of Michigan, Ann Arbor, MI 48109 USA; 13 VA Boston Healthcare System, Boston, MA USA; 14 Division of Cardiovascular Medicine, Department of Medicine, Brigham and Women’s Hospital, Boston, MA USA

**Keywords:** Computational biology and bioinformatics, Cardiovascular genetics

## Abstract

Spontaneous coronary artery dissection (SCAD) is a non-atherosclerotic cause of myocardial infarction (MI), typically in young women. We undertook a genome-wide association study of SCAD (N_cases_ = 270/N_controls_ = 5,263) and identified and replicated an association of rs12740679 at chromosome 1q21.2 (*P*_discovery+replication_ = 2.19 × 10^−12^, OR = 1.8) influencing *ADAMTSL4* expression. Meta-analysis of discovery and replication samples identified associations with *P* < 5 × 10^−8^ at chromosome 6p24.1 in *PHACTR1*, chromosome 12q13.3 in *LRP1*, and in females-only, at chromosome 21q22.11 near *LINC00310*. A polygenic risk score for SCAD was associated with (1) higher risk of SCAD in individuals with fibromuscular dysplasia (*P* = 0.021, OR = 1.82 [95% CI: 1.09–3.02]) and (2) lower risk of atherosclerotic coronary artery disease and MI in the UK Biobank (*P* = 1.28 × 10^−17^, HR = 0.91 [95% CI :0.89–0.93], for MI) and Million Veteran Program (*P* = 9.33 × 10^−36^, OR = 0.95 [95% CI: 0.94–0.96], for CAD; *P* = 3.35 × 10^−6^, OR = 0.96 [95% CI: 0.95–0.98] for MI). Here we report that SCAD-related MI and atherosclerotic MI exist at opposite ends of a genetic risk spectrum, inciting MI with disparate underlying vascular biology.

## Abstract (web summary)

Spontaneous coronary artery dissection (SCAD) is a cause of myocardial infarction Here, the authors present a genome-wide association study of SCAD, finding an association at 1q21.2 which potentially affects expression of ADAMTSL4.

## Introduction

Spontaneous coronary artery dissection (SCAD) is an increasingly recognized cause of myocardial infarction (MI) in young and otherwise healthy women, for which the etiology is incompletely understood. SCAD is an important cause of MI in women aged <50 years^1^. SCAD is defined as a non-traumatic, non-iatrogenic, and non-atherosclerotic separation of the coronary arterial wall by intramural hemorrhage, most often elicited by spontaneous intimal tear or rupture of vasa vasorum, causing accumulation of intramural hematoma that compresses the true arterial lumen, resulting in compromised coronary artery blood flow and MI (Supplementary Fig. 1). The current hypothesis is that SCAD results from a combination of susceptibility to dissection due to a predisposing arteriopathy that weakens the arterial wall, compounded by an additional precipitating trigger (i.e., emotional or physical stressor) that culminates in the arterial disrupion^2^.

The most common arteriopathy reported to co-occur with SCAD is fibromuscular dysplasia (FMD)^2–8^. Arterial dissections were reported in ~26% of individuals with FMD^9^, including SCAD in 2.7% of patients in the updated US FMD registry^9^. FMD is familial in some cases, with autosomal-dominant inheritance pattern and incomplete penetrance^10–13^. Familial studies of SCAD inheritance are lacking, although familial clustering has been observed^14,15^. A common variant on chromosome 6p24.1 in the *PHACTR1* gene, rs9349379-A (minor allele frequency (MAF) ~0.4), has been associated with both FMD and SCAD (odds ratio (OR)_FMD_ = 1.4, OR_SCAD_ = 1.7)^13,16^. Notably, the same allele has also been associated with cervical artery dissection and migraine^17–20^, suggesting a common underlying genetic architecture. Less commonly observed in patients with SCAD are systemic inflammatory diseases in 5–12% (e.g., systemic lupus erythematosus, Crohn’s disease)^1,2^ and monogenic vascular connective tissue diagnoses in <5% of cases (e.g., Marfan syndrome due to *FBN1* pathogenic variation or vascular Ehlers–Danlos syndrome due to *COL3A1* pathogenic variation)^21,22^. The pathophysiology of SCAD may also be linked to female reproductive hormonal exposure, supported by the observation that 90% of SCAD cases occur in women, especially those who are young or middle aged^1,2,23^.

Using a genome-wide association study (GWAS) approach, we identify multiple risk loci for SCAD and extend the relevance of the GWAS findings to an association with SCAD risk in a cohort of 412 individuals with FMD, as well as MI risk in more general populations through analysis of the UK Biobank (UKB) and Million Veteran Program (MVP).

## Results

### Phenotypic characteristics of the SCAD cohort

The SCAD discovery analysis was performed using samples from the Canadian SCAD (CanSCAD) Study (*N*_cases_ = 272)^24^. Each participant’s SCAD diagnosis was verified by coronary angiography and adjudicated by a core angiographic laboratory. Consistent with prior descriptions, 89% of the discovery sample was female, 2.6% of cases presented during the peripartum period, and the mean age of individuals presenting with SCAD was 53.2 ± 9.7 years. Upon review of the medical record, genetics referrals, and genetic testing results, one individual was found to have Loeys–Dietz syndrome with a pathogenic variant in *TGFBR2* (c.1591G>A, p.Ala531Thr), and one individual had a clinical diagnosis of Marfan Syndrome. FMD was identified in 60.9% of SCAD cases. The demographics and clinical characteristics of the discovery sample are summarized in Supplementary Table 1.

### GWAS of SCAD

**Fig. 1:**
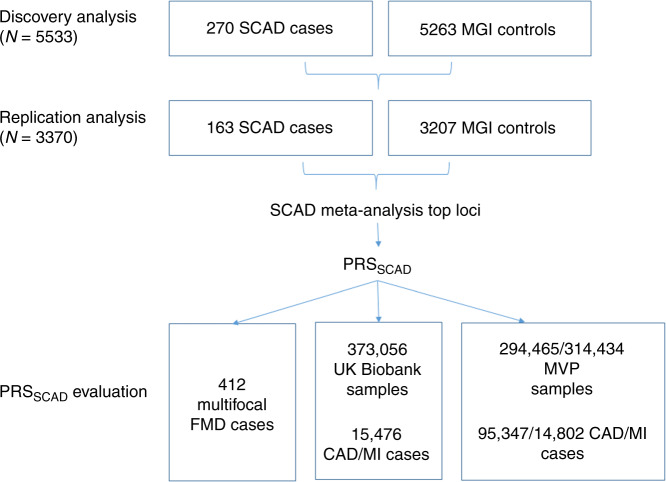
Discovery study design for the SCAD genome-wide association analysis (GWAS) and PRS_SCAD_ development and testing. In both the discovery and replication phases, CanSCAD study samples were analyzed with control subjects derived from the MGI biorepository with electronic health record-based phenotyping to exclude individuals with vascular diseases or connective tissue disorders. The association of genetic variants with SCAD and MI was tested by means of a PRS developed with the top ranked loci in the GWAS meta-analysis of SCAD discovery and replication analyses (PRS_SCAD_). The PRS_SCAD_ was tested for association with SCAD and MI in a cohort of individuals with FMD and for CAD and MI in the UK Biobank and MVP.

To discover genetic variants influencing the risk of SCAD, we undertook a GWAS of SCAD, utilizing samples from the CanSCAD Study and control subjects without vascular disease from the Michigan Genomics Initiative (MGI) biorepository (Fig. 1). In order to maximally leverage the biorepository resource and analyze the largest sample size possible, we employed the SAIGE method to conduct association analyses accounting for imbalanced case-to-control ratios^25^. The discovery analysis was comprised of 270 successfully genotyped SCAD samples from individuals enrolled between 2017 and 2018, after excluding 2 individuals with genetic syndrome diagnoses. Using MGI demographic data and principal component analysis (PCA), we identified age, sex, and ancestry-matched control subjects in the MGI biorepository of >50,000 individuals who had provided consent for genetic study and access to electronic health records (EHRs) and who did not have vascular disease or connective tissue disorder diagnoses. Ancestry estimation of our SCAD cases was performed by plotting PCs against world-wide Human Genome Diversity Project (HGDP) groups. The majority of discovery SCAD samples were predominantly European ancestry, and 9% were either mixed or predominantly East Asian ancestry (Supplementary Fig. 2). Up to 21 age-, sex-, and PCs-matched controls were selected for each SCAD case (*N*_controls_ = 5263). Genotyping of both CanSCAD and MGI samples was successfully performed using the Illumina Human CoreExome BeadArray v1.1 genotyping array, with 607,778 genotyped variants. Genotype quality control (QC) and imputation to the Haplotype Reference Consortium (HRC)^26^ reference panels were performed on case and control samples together, and 6,690,240 variants (imputation *r*-squared ≥ 0.8 and MAF ≥ 1%) were analyzed in 5533 case and control samples in the discovery analysis.

Our SCAD GWAS identified rs12740679 at chromosome 1q21.2 to be significantly associated with SCAD (*P* = 2.9 × 10^−10^, MAF = 0.26, OR = 1.97 [95% confidence interval (CI): 1.60–2.43]; Table 1, Supplementary Data 1). A sensitivity analysis confirmed no significant ancestry-specific effect of including individuals of East Asian ancestry in the discovery analysis. We compared GWAS results after removing individuals of Asian ancestry to GWAS results after removal of a comparable number of individuals of non-Asian ancestry from the discovery GWAS, over 10 iterations. In all analyses, the chromosome 1q21.2 locus demonstrated *P* < 5 × 10^−8^ and the distribution of results was not different between the removal of individuals of Asian ancestry as compared to the removal of individuals of non-Asian ancestry (permutation *P* = 0.4). We next conducted a replication analysis in similarly acquired samples from the CanSCAD study (*N* = 163) of individuals enrolled from 2018 to 2019. The replication samples were genotyped on the same platform, matched to unique MGI control subjects by age, sex, and PC-estimated ancestry that had not been previously included in the discovery GWAS (*N*_controls_ = 3207). QC and imputation to the HRC reference panel were performed on replication case and control samples together. The chromosome 1q21.2 locus was replicated in the independent analysis (OR = 1.56 [95% CI: 1.21–2.02], *P* = 7.32 × 10^−4^; Table 1, Supplementary Data 1).

**Table 1:** GWAS meta-analysis associations with *P* < 5 × 10^−8^.

Locus	rsID	Gene	Effect allele	Discovery (*N* = 5533)^a^				Replication (*N* = 3370)^b^				Meta-analysis				
				OR [95% CI]	*β*	s.e.	*P*	OR [95% CI]	*β*	s.e.	*P*	OR [95% CI]	*β*	s.e.	*P*	EAF
SCAD meta-analysis																
1q21.2	rs12740679	Near *C1orf51*	G	1.97 [1.60–2.43]	0.68	0.11	2.88E−10	1.56 [1.21–2.02]	0.45	0.13	7.32E−04	1.80 [1.53–2.12]	0.59	0.08	2.19E−12	0.26
6p24.1	rs9349379^c^	*PHACTR1*	A	1.54 [1.28–1.84]	0.43	0.09	4.14E−06	1.43 [1.13–1.81]	0.36	0.12	2.70E−03	1.50 [1.29–1.73]	0.40	0.07	4.36E−08	0.62
12q13.3	rs11172113^c^	*LRP1*	T	1.52 [1.26–1.83]	0.42	0.09	1.05E−05	1.50 [1.19–1.90]	0.41	0.12	6.77E−04	1.51 [1.31–1.75]	0.41	0.07	2.63E−08	0.61
21q22.11	rs28451064	Near *LINC00310*	G	1.71 [1.29–2.28]	0.54	0.15	2.10E−04	1.99 [1.40–2.83]	0.69	0.18	1.25E−04	1.82 [1.46–2.27]	0.60	0.11	1.19E−07	0.87

Locus	rsID	Gene	Effect allele	Discovery (*N* = 4895)^d^				Replication (*N* = 2996)^e^				Meta-analysis				
				OR [95% CI]	*β*	s.e.	*P*	OR [95% CI]	*β*	s.e.	*P*	OR [95% CI]	*β*	s.e.	*P*	EAF
SCAD females-only meta-analysis																
1q21.2	rs12740679	Near *C1orf51*	G	2.07 [1.65–2.59]	0.73	0.11	2.78E−10	1.51 [1.15–1.98]	0.41	0.14	3.03E−03	1.82 [1.53–2.16]	0.60	0.09	1.48E−11	0.26
6p24.1	rs9349379^c^	*PHACTR1*	A	1.55 [1.27–1.88]	0.44	0.10	1.15E−05	1.38 [1.08–1.77]	0.32	0.13	1.10E−02	1.48 [1.27–1.72]	0.39	0.08	5.13E−07	0.62
12q13.3	rs11172113^c^	*LRP1*	T	1.56 [1.28–1.90]	0.44	0.10	1.18E−05	1.51 [1.17–1.93]	0.41	0.13	1.29E−03	1.54 [1.32–1.80]	0.43	0.08	5.59E−08	0.61
21q22.11	rs28451064	Near *LINC00310*	G	1.84 [1.35–2.50]	0.61	0.16	9.28E−05	2.13 [1.47–3.10]	0.76	0.19	7.56E−05	1.95 [1.54–2.47]	0.67	0.12	3.20E−08	0.87

Results of the overall SCAD GWAS meta-analysis (total *N* = 8903), including both males and females, and the females-only GWAS meta-analysis (total *N* = 7891) are shown. Discovery GWAS and replication association analysis were all based on generalized mixed models in SAIGE, which uses the saddlepoint approximation (SPA) correction that accounts for case and control imbalances. GC correction was applied before standard error-weighted meta-analysis. SNPs with imputation Rsq ≥ 0.8 and MAF ≥ 1% were analyzed. *P* values here are two sided and unadjusted from multiple correction. Variants with association *P* < 5 × 10^−8^ pass the genome-wide significance Bonferroni-corrected threshold. All of the association models are adjusted for the first 5 PCs; and age, sex, and PCs matched between cases and controls.

*Rsq* *r*-squared, *OR* odds ratio, *CI* confidence interval, *s.e.* standard error, *EAF* effect allele frequency, *AF* allele frequency.

^a^270 cases vs. 5263 ctrls, *λ*_GC_ = 0.94.

^b^163 cases vs. 3207 ctrls, *λ*_GC_ = 0.97.

^c^Genotyped SNP.

^d^241 case vs. 4654 ctrls, *λ*_GC_ = 0.97.

^e^146 case vs. 2850 ctrls, *λ*_GC_ = 0.96.

Meta-analysis of the genome-wide SCAD discovery and replication results identified additional genome-wide significant associations at the chromosome 12q13 *LRP1* locus (rs11172113) and the chromosome 6p24.1 *PHACTR1* locus (rs9349379) (Table 1, Supplementary Data 1, Fig. 2, Supplementary Data 2). The chromosome 6p24.1 association was consistent with a previously published association with SCAD of similar magnitude, with an OR of 1.5 in the current study compared to an OR of 1.8 reported previously^16^. Conditional analyses based on meta-analysis of individual conditional results of discovery studies and replication studies were performed on the top loci with a false discovery rate (FDR) *q* value < 0.05, which corresponded to *P* < 5 × 10^−6^, for follow-up analysis using a polygenic risk score (PRS) approach. This review yielded seven independent loci (rs11207415, rs12740679, rs78377252, rs9349379, rs78349783, rs11172113, rs28451064; Supplementary Table 2). A secondary GWAS meta-analysis of only females in the main SCAD discovery and replication GWAS analyses (*N* = 7891) showed similar findings in the top chromosome 1q21.2 locus with *P* < 5 × 10^−8^, as well as a new association at chromosome 21q22.11 (rs28451064) (OR = 1.95 [95% CI: 1.54–2.47], *P* = 3.2 × 10^−8^; Table 1, Supplementary Data 1, Supplementary Fig. 3). A secondary analysis of SCAD with and without FMD was conducted separately (*N* = 2922 and *N* = 2655, respectively), confirming that no additional signals were identified, and the results of the top three loci from the overall GWAS meta-analysis are largely consistent and noted to be ranked highly in the SCAD+FMD GWAS (Supplementary Table 3). The top chromosome 1q21.2 association results in the main GWAS and secondary analyses demonstrated association of SCAD with this locus regardless of FMD status (Supplementary Table 4), although not meeting a genome-wide significance threshold of *P* < 5 × 10^−8^, likely due to reduced sample size and loss of statistical power of these analyses. We used the discovery stage genotypes to estimate the heritability of SCAD, and the estimated genetic proportion of variance explained (PVE) was 0.26 (standard error 0.045).

**Fig. 2:**
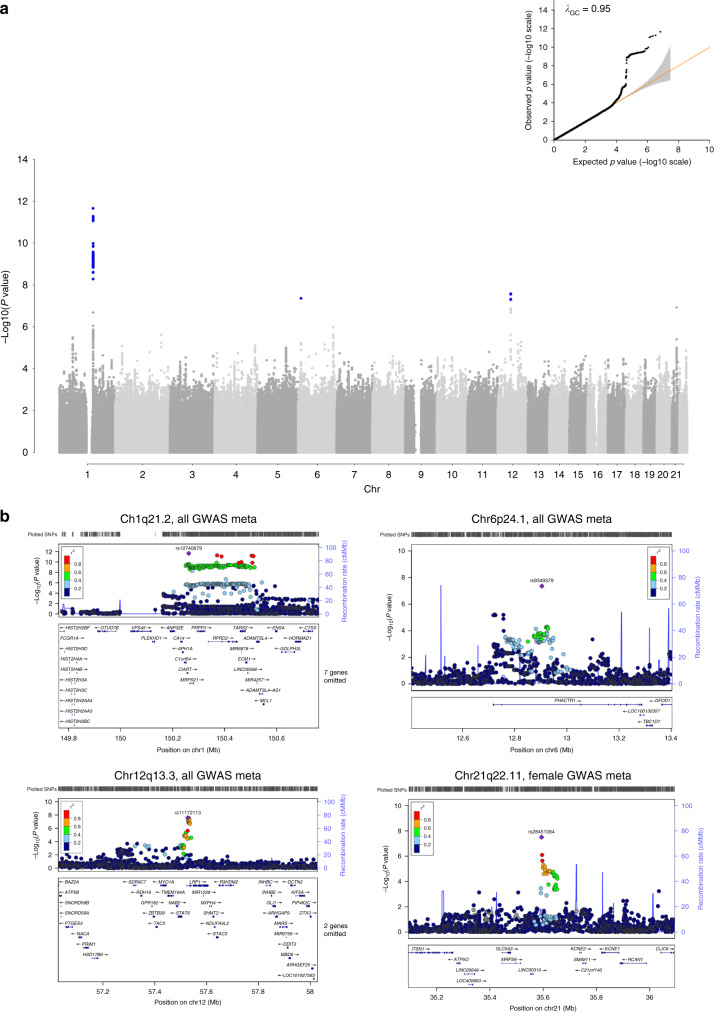
SCAD GWAS meta-analysis results. **a** Manhattan plot and QQ plot for the meta-analysis of SCAD discovery and replication groups (*N*_cases_ = 433, *N*_controls_ = 8470). Discovery GWAS and replication association analysis were all based on generalized mixed models in SAIGE, which uses the saddlepoint approximation (SPA) correction that accounts for case and control imbalances. GC correction was applied before standard error-weighted meta-analysis. SNPs with imputation Rsq ≥0.8 and MAF ≥1% were analyzed. *P* values are two sided and unadjusted for multiple testing and variants meeting the genome-wide significance Bonferroni-corrected threshold (association *P* < 5 × 10^−8^) are shown in blue. All of the association models are adjusted for the first five PCs, and age, sex, and PCs matched between cases and controls. The *λ*_GC_ value is 0.95. **b** Regional association plots with gene annotation for the chromosomes 1q21, 6p24, and 12q13 loci are shown with the index SNP and additional SNPs within 500 Kbp in each direction in the meta-analysis of SCAD GWAS discovery and replication groups. The association test used was the same as **a**. Similarly, the chromosome 21q22.11 locus region identified in the GWAS meta-analysis of female subjects is shown. LD *r*^2^ and recombination rate information were estimated based on 1000G EUR population.

### Transcript expression analyses for quantitative trait loci (QTLs) and sex differences

**Fig. 3:**
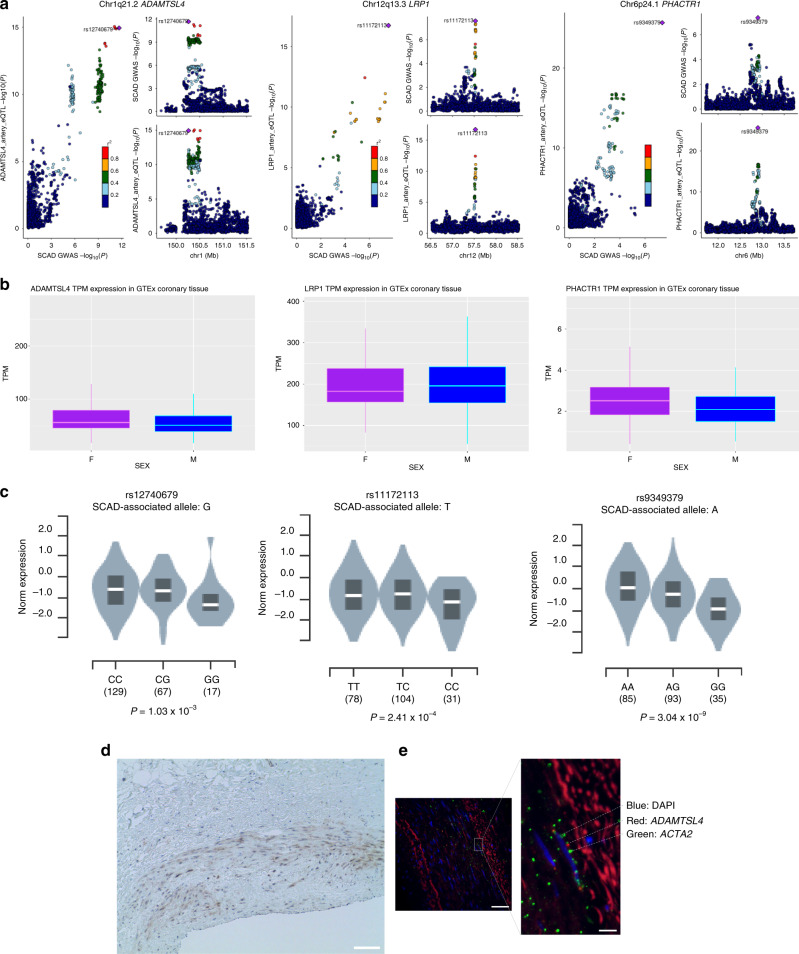
Arterial expression of genes implicated by the SCAD GWAS meta-analysis. **a** Colocalization analysis results in which the lead SNP identified matched the queried transcript are shown for each locus identified. The locus-compare scatter plot compares linear regression eQTL (*n* = 913) and GWAS results (*n* = 8903), using the same method as Fig. 2a, in the gene region, which indicates whether the GWAS top locus is also the leading SNP in the eQTL result, supporting the conclusion that both traits are associated and share a single causal variant. *P* values are two sided and unadjusted for multiple testing. The gene prioritized in each locus is shown on the *y*-axis and corresponding figure label. **b** Boxplots of transcript expression levels by sex (*n* = 913, which includes 593 males and 320 females) are displayed for each gene prioritized by the colocalization analysis. Transcript expression levels are measured in transcripts per million (TPM). Gene TPMs were downloaded from the GTEx portal (v7) and subset to include only values from coronary artery tissue. The median of each gene is represented as the center horizontal line within each box, colored light purple for females and light blue for males. The top boundary of the box for each gene represents the 75th percentile of associated TPM values and the bottom boundary of the box represents the 25th percentile. The end point of the whiskers extending from each box mark the minimum and maximum TPM values, respectively. **c** Expression QTL results in GTEx arterial tissues. Violin plots depict normalized expression by allele. Two-sided values of linear regression *P* values listed represent calculated association between genotypes of each listed SNP and the corresponding eGene of interests referenced in **a**, **b**. **d** Arterial ADAMTSL4 immunostains of normal human coronary artery showing staining (brown) in the arterial media (×20), with nuclei stained blue. Inset bar = 100 µm. **e** In situ hybridization of normal human coronary artery with *ADAMTSL4* mRNA (red) detected in the arterial media and with alpha actin (green) with nuclear DAPI staining (blue) co-localization to smooth muscle cells (×40, inset bar = 50 µm) and magnified inset (inset bar = 10 µm).

We hypothesized that the functional gene(s) at the chromosome 1q21.2 locus would have expression levels regulated by the index variant rs12740679 as an expression QTL (eQTL) that would be identified in a colocalization analysis and that the regulated gene(s) would be expressed in vascular tissue and smooth muscle cells. We used the Genotype-Tissue Expression (GTEx) portal^27^ to examine the mRNA expression of genes within the 500 Kb surrounding the noncoding rs12740679 locus and identified the most significant association in the colocalization analysis in arterial tissues (coronary artery, tibial artery, and aorta) for *ADAMTSL4* (Fig. 3a), with eQTL linear regression *P*_aorta_ = 2.6 × 10^−17^ and *P*_tibial artery_ = 2.0 × 10^−25^ (Supplementary Data 3). Coronary artery expression demonstrated wider variability in expression value, with an eQTL association of *P*_coronary_ = 1.03 × 10^−3^ (Fig. 3c). *ADAMTSL4* mRNA was strongly expressed in arterial tissues, as well as other organs comprised of smooth muscle (Supplementary Fig. 4). In order to localize ADAMTSL4 protein and mRNA expression, we performed immunostaining and in situ hybridization (Fig. 3d, e); these studies both demonstrated expression in the arterial media smooth muscle cells, consistent with the location of arterial disruption in SCAD. The expression of *ADAMTSL4* in arterial tissues was 1.1-fold higher in women as compared to men across all arterial tissues in GTEx (Wald test *P* = 1.3 × 10^−5^; Fig. 3b, Supplementary Data 3). In the chromosomes 6p24.1 and 12q13.3 loci, rs9349379 and rs11172113 were each identified as significant eQTLs in arterial tissues regulating the expression of *PHACTR1* and *LRP1*, respectively (Fig. 3a–c, Supplementary Data 3), with *PHACTR1* mRNA expressed 1.2-fold higher in the coronary arteries of women (Wald test *P* = 8.2 × 10^−3^; Supplementary Data 3). In the chromosome 21q22.11 locus, rs28451064, although no genes passed the threshold of 75% posterior probability, *MRP6* and *KCNE2* were identified as the top transcripts in the colocalization analysis. *MRP6* is expressed in the arteries (Supplementary Data 3, Supplementary Fig. 5) but showed no differences in expression level according to sex (Supplementary Data 3).

### Analysis of a SCAD polygenic risk score (PRS_SCAD_) in an at-risk FMD cohort

Arterial dissections occur in a subset of individuals with multifocal FMD (mFMD) and may lead to substantial morbidity due to organ hypoperfusion and ischemia, with clinical manifestations including MI due to SCAD. Given the overlap of FMD and SCAD diagnoses in some individuals, we hypothesized that alleles discovered by our GWAS would be associated with SCAD events in individuals with mFMD and tested this in a cohort of individuals with mFMD consecutively enrolled at presentation to subspecialty clinics with angiographic evidence of mFMD. As a confirmation analysis of the chromosome 1q21.2 association with SCAD, we performed a case–control GWAS using the FMD cohort cases and age-, sex-, and ethnicity-matched controls from the Cleveland Clinic Genebank, which showed replication at this locus (OR = 1.92 [95% CI: 1.06–3.45], *P* = 0.032, GWAS *λ*_GC_ = 0.98). Next, we tested whether the top-ranked alleles identified in the SCAD GWAS meta-analysis were predictive of SCAD in the FMD cohort. Among the 412 unrelated subjects, 131 subjects had at least one arterial dissection, and 281 subjects had no documented arterial dissections; 28 unrelated individuals had experienced SCAD. Applying a PRS developed from the top-ranked SCAD GWAS meta-analysis loci with FDR *q* value <0.05 in the SCAD meta-analysis (PRS_SCAD_), the PRS_SCAD_ was associated with increased SCAD risk in a weighted continuous logistic regression model (OR = 1.82 per 1 SD unit, 95% CI [1.09–3.02], *P* = 0.021, Supplementary Table 5). The PRS_SCAD_ result appears to be driven primarily by an association of rs11207415 on chromosome 1p32.1 (OR = 2.09 [95% CI: 1.21–3.63], *P* = 9.36 × 10^−3^) that is independent of the top SCAD-associated variant, rs12740679 at chromosome 1q21.2 (Supplementary Data 4). Finally, we conducted as a secondary analysis a vascular phenome-wide association study (“vascular PheWAS”) in the FMD samples fully independent of the SCAD GWAS meta-analysis, to comprehensively assess the occurrence of arterial aneurysm, dissection, and stenotic lesions of FMD, by testing the association of the PRS_SCAD_ with each vascular finding using a continuous logistic regression (Supplementary Table 5), showing nominal associations (*P* < 0.05) with cervical artery mFMD (OR = 1.58 [95% CI: 1.13–2.22] per 1 SD unit, *P* = 0.0079) and an inverse association with hypertension (OR = 0.74 [95% CI: 0.57–0.97] per 1 SD unit, *P* = 0.029) (Supplementary Table 5). These results suggest that the alleles in the PRS_SCAD_ predispose to fragility of the coronary arterial wall, even among those already affected with FMD.

### Pleiotropy of the SCAD-associated risk loci

The chromosome 6p24.1 *PHACTR1* locus rs9349379-A allele has been associated with SCAD, FMD, cervical artery dissection, migraine headache, and hypertension, and the rs9349379-G allele has been associated with coronary artery disease (CAD) and MI more typically due to atherothrombotic mechanisms and more frequently occurring in men^13,16,17,28,29^. All three of the discovered loci in the SCAD GWAS meta-analysis have been described in association with migraine headache, which is observed in 32.3% of patients with FMD^30^ and 32.9% of the CanSCAD cohort (Supplementary Table 1, Supplementary Data 5). Using risk scores based on CAD-associated single-nucleotide polymorphisms (SNPs; 386 SNPs derived from the GWAS Catalog^31^), we observed a strong inverse relationship between the score, with genetically increased CAD risk conferring a protective effect from SCAD (OR = 0.78 per 1 SD unit increase, 95% CI: 0.68–0.89, *P* = 3.23 × 10^−4^), and this was robust to the removal of the chromosome 6p24.1 locus from the risk score (OR = 0.82 per 1 SD unit increase, 95% CI: 0.71–0.94, *P* = 3.75 × 10^−3^) with several individual SNPs showing nominal association (*P* < 0.05) with opposing direction of effect between CAD and SCAD (Supplementary Data 6, Supplementary Table 6).

### Analysis of the PRS_SCAD_ in the UKB and MVP

**Fig. 4:**
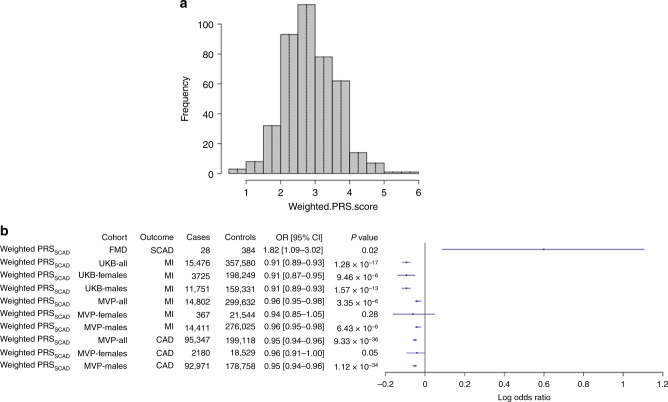
PRS_SCAD_ and prevalent SCAD and MI events in an FMD cohort and the UK Biobank. **a** Histogram of the weighted PRS_SCAD_ based on FMD cohort (*N* = 412) with overlaying the corresponding data points in each interval. **b** The ORs and corresponding 95% CIs are shown for the association of PRS_SCAD_ with SCAD-MI, all MI, or CAD/MI, in the FMD cohort (*n* = 412, which includes 28 SCAD cases), UK Biobank (*n* = 373,056, which includes 15,476 CAD/MI cases), and MVP (*n* = 294,465, which includes 95,347 CAD cases; *n* = 314,434, which includes 14,802 MI cases). The logistic regression Wald statistic of two-sided *P* values are displayed. All models are adjusted by age, sex, and PCs. *P* values are unadjusted for multiple testing.

**Fig. 5:**
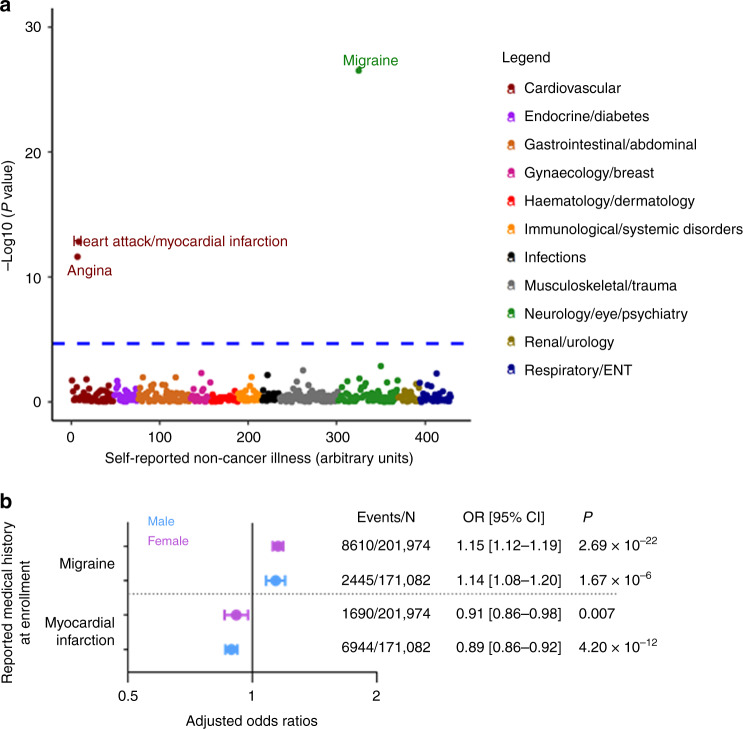
PheWAS phenotypic associations in the UK Biobank. **a** PheWAS of the PRS_SCAD_ vs. self-reported non-cancer medical illnesses in the UK Biobank (*n* = 373,015). The different color dots represent the association results (logistic regression two-sided test *P* values in minus log base ten scale) of the PRS_SCAD_ for non-cancer illness categories. Two-sided *P* values were considered significant when below a Bonferroni-adjusted threshold (0.05/2356 ≈ 2.12 × 10^−5^). **b** Sex-specific ORs in blue circles for males (*n* = 171,082) or purple circles for females (*n* = 201,974) are shown in the center of the corresponding 95% confidence interval (CI) from the PRS_SCAD_ analysis for migraine headache and MI, with two-sided values of *P* values displayed. Events in the legend denote the number of migraine cases or MI cases among the female or male groups, with *N* shown for the total sample size for each group. The models were adjusted for age at enrollment, genetic sex, genotyping array and batch, and the first four PCs. The logistic regression Wald statistic of two-sided *P* values are displayed.

We further assessed pleiotropy of the SCAD-associated loci in the UKB published results (http://geneatlas.roslin.ed.ac.uk/phewas/; PheWAS^32^), demonstrating associations of chr6p24.1 rs9349379-G (*PHACTR1*) and chr21q22.11 rs28451064-A (*MRP*6*/KCNE2*) with CAD (Supplementary Data 5). There was insufficient phenotyping and/or too few events of SCAD and MI in women aged <50 years to replicate the associations with SCAD in the UKB. Of the SNPs included in the PRS_SCAD_, two SNPs had at least nominal association with atherosclerotic MI risk (Supplementary Data 4) at chromosome 6p24.1 (rs9349379) and chromosome 21q22.11 (rs28451064), with each locus having a directionally opposite effect for SCAD. The effect estimate of the inverse association of the PRS_SCAD_ with MI was comparable between men (adjusted hazard ratio (HR) = 0.91 per 1 SD unit increase, 95% CI [0.89–0.93], *P* = 1.57 × 10^−13^) and women (adjusted HR = 0.91 per 1 SD unit increase, 95% CI [0.87–0.95], *P* = 9.46 × 10^−6^) (Fig. 4, Supplementary Table 7), with a 3.72-fold higher MI event rate in men (*N* = 11,751/171,082) as compared to women (*N* = 3725/201,974). There was no significant sex interaction with the PRS_SCAD_. We further replicated in the MVP the inverse association of the PRS_SCAD_ with CAD risk (OR = 0.95 per 1 SD unit increase, 95% CI [0.94–0.96], *P* = 9.33 × 10^−36^) and MI risk (OR = 0.96 per 1 SD unit increase, 95% CI [0.95–0.98], *P* = 3.35 × 10^−6^) and also without substantial differences in effect estimates between men and women (Supplementary Table 8). Both the UKB and MVP analyses were repeated after removing the chromosome 6p24.1 locus that is already known to have inverse risk for CAD/MI and SCAD, and the results remained comparable and statistically significant (Supplementary Tables 7 and 8). Thus the UKB and MVP results both robustly support that there are opposing mechanisms between SCAD-related MI and atherosclerotic MI/CAD, involving the top-ranked loci identified in our SCAD GWAS meta-analysis (Fig. 4b, Supplementary Table 6). Finally, a PheWAS of UKB data highlighted that a weighted and continuous PRS_SCAD_ was associated with MI, migraine headache, medications used to treat migraine headache, tinnitus, and coronary artery revascularization, with concordant ORs in men and women for both migraine headache and MI (Fig. 5, Supplementary Fig. 6, Supplementary Data 7).

## Discussion

Using a GWAS approach, we identified an association of rs12740679 at the chromosome 1q21.2 locus, implicating the extracellular matrix protein-encoding gene *ADAMTSL4*. We additionally replicated a previously reported association of an intronic variant in the *PHACTR1* gene, at chromosome 6p24.1, and identified associations of non-coding variants in the *LRP1* gene at chromosome 12q13.3 and near *MRP6/KCNE3* at chromosome 21q22.11. To explore pleiotropy and biologic underpinnings of SCAD and related arterial diseases, we developed and tested a genetic risk score, based on the SCAD GWAS findings. These analyses demonstrated association with SCAD occurrence in an independent FMD cohort analysis, as well as opposing risks of atherosclerotic-related MI and SCAD-related MI, in two large cohort studies, UKB and MVP. Further, the score was concordantly associated with increased risk of migraine headache and tinnitus, a clinical feature of FMD, in the UKB population. Notably, the association of the SCAD risk score supports a hypothesis previously suggested by the *PHACTR1* locus association patterns^16^, of sex-dimorphic and opposing influences on vascular biology, whereby one end of the spectrum of risk leads to arterial fragility and predisposition to arterial dissection with resulting MI, predominantly in women, and the other end of the spectrum of risk contributes to susceptibility to coronary atherosclerotic MI, a disease that affects both sexes but occurs more often in men.

Until recently, SCAD was rarely diagnosed, and little was known about the relevant vascular biology. Increased recognition of the disease, particularly in young women, and modern coronary angiographic methods have led to improved diagnoses, such that SCAD is now recognized as an important cause of MI in women aged <50 years^1^. Owing to the female preponderance and peripartum occurrence in a small subset (<5%) of cases, hormonal factors have been implicated, but with little mechanistic data to support a specific role. SCAD and mFMD have overlapping phenotypes, both occurring predominantly in women (9:1 ratio of women to men with both diagnoses), and approximately 61% of our SCAD discovery cohort had mFMD. Both SCAD and FMD typically occur in individuals without a high burden of traditional risk factors for atherosclerosis, such as hypertension, hyperlipidemia, smoking, or diabetes. While luminal stenosis of the coronary arteries is not commonly observed in FMD, coronary arterial wall abnormalities have been documented^33^. FMD is currently understood as a likely genetically heterogeneous condition with both sporadic and familial forms, with at least a partially complex genetic basis. While SCAD may occur in individuals with monogenic conditions such as Marfan Syndrome (due to *FBN1* pathogenic variants), Loeys–Dietz syndrome (due to *TGFBR1/2* and other transforming growth factor-β pathway gene variants), and vascular Ehlers–Danlos syndrome (due to *COL3A1* variants), this is uncommon (<5%); no such molecular diagnoses have been defined for FMD.

The chromosome 1q21.3 locus regulates the arterial expression of the gene prioritized by colocalization analysis in this region, *ADAMTSL4*. *ADAMTSL4* is a member of the ADAMTS (a disintegrin and metalloproteinase with thrombospondin motifs)-like gene family, and it encodes an extracellular matrix protein that binds to fibrillin-1 to promote the formation of microfibrils in the matrix^34^. *ADAMTSL4* pathogenic variants underlie an autosomal-recessive form of ectopia lentis^35–37^, and fibrillin-1 gene variants may cause autosomal-dominant ectopia lentis and Marfan Syndrome^38^ Our histopathologic data localizing ADAMTSL4 protein and mRNA expression to the medial layer of the arterial wall, and medial vascular smooth muscle cells specifically, are consistent with the arterial media as the site of dissection and intramural hemorrhage^4^. That women demonstrate higher basal expression of *ADAMTSL4*, and the allele conferring risk for SCAD is associated with lower expression of this gene, implicates a hypothesis of relative deficiency of ADAMTSL4 involved in a mechanism of promoting arterial fragility, as has been observed in disorders of fibrillin-1 deficiency^39^. Whether the pathogenic defect is insufficiency of the extracellular ADAMTSL4 protein itself, or potentially altered signaling events as has been described in the setting of *FBN1* pathogenic genetic variation^40^, will need to be defined in further, mechanistic studies.

The chromosome 6p24 locus (rs9349379-A) associated with FMD^13^ and SCAD^16^ is located at an enhancer in aortic tissue. Mechanisms have been suggested for regulation of phosphatase and actin regulator 1 (*PHACTR1*)^41^ and neighboring endothelin-1 (*EDN1*) gene transcription^42^. Low-density lipoprotein receptor-related protein (*LRP1*) encodes a cell membrane-associated protein that interacts with a number of secreted proteins and cell surface molecules to mediate their endocytosis or the activation of signaling pathways. *LRP1* GWAS-implicated genetic variants have been associated with migraine headache^18^ and abdominal aortic aneurysm^43^. Disruption of *Lrp1* in vascular smooth muscle cells in mice leads to loss of vascular wall integrity and increased susceptibility to atherosclerosis^44^. The chromosome 21q22.11 locus prioritized genes include multidrug resistance protein-6 (*MRP6*), also known as ATP-binding cassette subfamily C, member 6, or *ABCC6*, which encodes for a cellular transporter, and pathogenic variants inherited in a recessive pattern have been described to cause pseudoxanthoma elasticum, a connective tissue disorder with characteristic arterial dysplasia characterized by calcification in elastic tissues.

The current study’s findings support that SCAD, migraine headache, and FMD at least partly share a common genetic basis. Notably, the same SCAD-associated *PHACTR1* locus and *LRP1* locus alleles have been associated with cervical artery dissection^17^, which has been documented in 16.4% of individuals with FMD^9^, and migraine headache, which occurs in ~36% of individuals with SCAD^7^ and ~50% with FMD^9^. Despite the observed genetic pleiotropy of these loci, and that a substantial proportion of individuals with SCAD are also diagnosed with multifocal FMD, SCAD has been documented in only 2.7% of individuals with FMD^9^. As such, there is currently an unmet clinical need for risk stratification of individuals with FMD to identify the subset at risk for SCAD-MI. By applying the PRS_SCAD_ in a cohort of individuals with FMD, we identified an association of the PRS_SCAD_ with SCAD events. Potential clinical implications of identifying an individual with FMD who is at elevated SCAD risk include: (1) the consideration of antiplatelet therapy to prevent thrombotic complications in the event of a dissection, (2) a need for especially close blood pressure monitoring and control, (3) consideration of pregnancy risk, (4) specific behavioral recommendations to reduce arterial strain (i.e., avoidance of isometric exercises), (5) potential avoidance of certain medications, such as triptans that have vasoactive properties and are commonly used to treat migraine headache and fluoroquinolone antibiotics that increase the risk of aortic dissection^45^, and (6) whether pharmacologic therapy may provide benefit for primary SCAD prevention (i.e., beta-blockers, to reduce arterial shear stress)^46^ or may cause harm^47^.

A notable finding from this study is the inverse relationship of the PRS_SCAD_ with MI caused by more common atherothrombotic etiologies. The PRS_SCAD_ was protective against MI events in the UKB population in both men and women and in men in the MVP. The MVP analysis of women was underpowered due to low sample size of women, but the results were directionally consistent as compared to the results in men. Prior studies have shown that a lower PRS_CAD_ is associated with an increased risk of migraine headache^48^ and conversely that migraine-associated genetic variants associate with reduced risk of CAD^49^. Based on the genetic correlation between migraine and SCAD, these findings support our observation of an inverse relationship between SCAD and CAD risk with the PRS_SCAD_.

Limitations of the current study included the relatively limited sample size of SCAD cases. Increasing the sample size would be expected to identify further associated loci, albeit with lower effect sizes. Ascertainment of additionally genotyped samples with appropriately adjudicated SCAD cases status is limited in population studies, due to the challenges posed by underdiagnosis of SCAD, and non-uniform interpretation of angiographic data, which is inconsistently performed in young women presenting with features of MI. Additional independent GWAS efforts for SCAD will provide an opportunity to enhance genetic discovery through meta-analysis of GWAS results^50^. While SCAD is more common in younger women, it also occurs in men and older women. Phenotypic characterization utilizing coronary angiography and uniform interpretation methods are lacking in large population samples, and this is a major obstacle to understanding the true prevalence and genetic risk for SCAD. However, the protective effect we identified for MI in the UKB and MVP populations, predominantly induced by coronary artery atherothrombosis, is significant and supports a rationale for further mechanistic study of the function of the GWAS-implicated arterial wall biology. Given the known sex differences in SCAD and CAD susceptibility, we evaluated sex-specific effects of the PRS_SCAD_ and sex interactions, which did not show any significant associations; however, the power to assess this in the current study is limited. Notably, we did not identify signals in monogenic disease genes previously implicated in SCAD. Specific pathogenic variants are unlikely to be detected by GWAS or exome chip, or by clinical assessment alone, and will be ideally addressed in sequencing studies to identify low-frequency, deleterious coding variants. The inclusion of such genetic syndrome cases in GWAS approaches may be justified based on the observation that SCAD occurs infrequently even among individuals with these diagnoses; thus the identified common variants influencing SCAD risk may still be relevant. Further subset analyses of the subgroup of patients with both SCAD and connective tissue disorder or monogenic arterial disease will require larger sample sizes in future research. PRS have been proposed as clinical tools for risk stratification in the population. Our PRS_SCAD_ may have such future utility but would require testing and validation in a population sample with accurate SCAD phenotyping in a sufficient number of individuals. Similarly, the clinical utility of the PRS_SCAD_ in individuals with FMD requires further validation.

In summary, we identified genetic associations for SCAD, an uncommon but important cause of MI, particularly in young women. The findings of the current study support a complex genetic basis of SCAD and new biological leads for further mechanistic investigation of arterial pathobiology and sex-dimorphic mechanisms. Importantly, the SCAD-associated alleles are associated with excess risk of SCAD among individuals with FMD, a partly overlapping arteriopathy with an elevated but poorly understood risk for arterial dissections. Our pleiotropy analyses support a shared genetic architecture for SCAD and migraine headache, as well as vascular effects predisposing to SCAD-associated MI, which are protective from atherosclerotic MI. These findings support a hypothesis of vascular pleiotropy with sex-dimorphic effects and warrant further investigation to understand targets for mitigating MI risk due to diverse vascular pathobiology.

## Methods

### Clinical samples

The current study is a prospective genetic study evaluating the genetics of SCAD in the large prospective cohort of patients enrolled in the CanSCAD Study. The CanSCAD study included SCAD patients from the prospective CanSCAD Cohort Study (funded by the Canadian Institutes of Health Research) and the Non-Atherosclerotic Coronary Artery Disease Study. Patients presenting with acute SCAD were prospectively enrolled from 22 sites throughout North America (20 sites in Canada and 2 in the United States). SCAD diagnosis was confirmed on coronary angiography by the University of British Columbia (UBC) core laboratory research team and categorized according to previously established Saw classification^51,52^. Type 1 SCAD depicts contrast dye staining of arterial wall with multiple radiolucent lumen, with or without dye hang-up or slow contrast clearing from the lumen. Type 2 SCAD depicts diffuse and smooth narrowing that varies in severity; Type 2A describes the presence of normal arterial segments proximal and distal to dissection; Type 2B describes dissection that extends to distal tip of the artery. Type 3 SCAD depicts focal or tubular stenosis that appears similar to atherosclerosis. Intracoronary imaging with optical coherence tomography or intravascular ultrasound was performed at the discretion of the treating physicians to aid angiographic diagnosis. Detailed baseline demographics, targeted history for predisposing conditions and precipitating stressors, and laboratory screening for predisposing conditions were performed. Screening for FMD was recommended for all SCAD patients, and mFMD was defined according to consensus guidelines^23^. Patients were prospectively followed post-discharge at 1, 6, and 12 months and annually thereafter for 3 years for cardiovascular events. Research ethics board approvals were obtained at each site (UM: HUM00044507, UBC: H14-00968), and all patients provided informed consent for participation.

In the CanSCAD Genetics Substudy, site-specific research ethics board approvals for the study and individual patient consents were obtained. Genetic studies were performed on CanSCAD patients who provided informed consent. Collection of DNA was obtained through blood or saliva self-collection kit (Oragene-500 Kit, DNAGenotek). DNA was extracted according to the manufacturer’s instruction (DNAGenotek) as previously described^53^ and quantified using the Quant-iT PicoGreen assay (Life Technologies). DNA samples were normalized to a concentration of 50 ng/μl for genotyping. The processed DNA samples were batched and transferred to the University of Michigan for GWAS analysis.

Between 2010 and 2015, adult subjects with mFMD (*N*_unrelated_ = 412) were enrolled with IRB approval in the Cleveland Clinic FMD Biorepository. All participants provided informed consent and study activities were approved by the enrolling institution’s IRBs. Each research participant contributed either a blood or saliva sample via standard K^+^ EDTA blood collection tubes or commercial saliva collection kits (Oragene, DNAGenotek). DNA was isolated according to commercial kit protocols (Nucleospin Tissue (TakaraBio)), extracted according to the prepIT-L2P Extraction Kit (DNAGenotek) and quantified using the Quant-iT PicoGreen dsDNA Kit (ThermoFisher).

The MGI is a program that recruited participants while awaiting diagnostic, interventional, and surgical procedures. Participants provided a blood sample for genetic analysis and agreed to link their sample to their EHR and other sources of health information^54^. The current study’s analyses involved 13,756 individuals from MGI genotyped with the same version (v1.1) of the Illumina BeadArray genotyping platform as the SCAD and FMD cases at the University of Michigan DNA Sequencing Core Facility. Several International Classification of Diseases (ICD) codes corresponding to diagnoses of arterial aneurysm, dissection, and non-atherosclerotic dysplasia and stenosis were excluded, as well as connective tissue disorders (Supplementary Data 8).

The Cleveland Clinic GeneBank study is a sample repository generated from consecutive patients undergoing elective diagnostic coronary angiography or elective cardiac computed tomographic angiography with extensive clinical and laboratory characterization and longitudinal observation. Subject recruitment occurred between 2001 and 2006. Ethnicity was self-reported and information regarding demographics, medical history, and medication use was obtained by patient interviews and confirmed by chart reviews. All patients selected as controls were age and sex matched to the FMD cohort and had no evidence of CAD, defined as adjudicated diagnoses of stable or unstable angina, MI (adjudicated definition based on defined electrocardiographic changes or elevated cardiac enzymes), angiographic evidence of 50% stenosis of one or more major epicardial vessel, and/or a history of known CAD (documented infarction, coronary disease, or history of revascularization). All patients provided written informed consent prior to being enrolled in GeneBank, and the study was approved by the Institutional Review Board of the Cleveland Clinic.

The UKB recruited adults aged 40–69 years from across the United Kingdom between 2006 and 2010^55^. Participants were assessed at study at study enrollment via medical histories, physical exams, and biochemical measurements. Participant data are linked to hospital episode statistics that span from before study enrollment to March 31, 2017.

The MVP^56^ recruited active users of the Veteran Health Administration (VA) of any age from >60 VA Medical Centers nationwide since 2011 with current enrollment at >825,000. Informed consent is obtained from all participants to provide blood for genomic analysis and access to their full EHR data within the VA prior to and after enrollment. Imputed genetic information is available for up to 314,434 participants assigned to white-European ancestry using the HARE algorithm^57,58^. We used inpatient and outpatient ICD9/10 diagnostic and Current Procedural Terminology codes to identify subjects with clinical CAD either before enrollment going back to 2002 or after enrollment until mid-August 2018. An individual was classified as a case if he or she had ≥1 admission to a VA hospital with discharge diagnosis of acute myocardial infarction (AMI) or ≥1 procedure code for revascularization of the coronary arteries or ≥2 ICD codes for CAD (410–414) on ≥2 dates. Individuals with only one ICD code for CAD on one date and no discharge diagnoses for AMI or revascularization procedures were excluded from the analyses and remaining subjects were classified as controls. This algorithm identified up to 95,347 unrelated subjects with CAD and 199,118 unrelated controls, with 19,969 subjects being excluded due to ambiguous CAD status. We performed subgroup analysis involving the subset of cases with evidence of a hospitalization for MI. These cases were compared to all controls in our association analysis (*N* = 14,802 unrelated MI cases and *N* = 299,632 unrelated controls).

### Genotyping on the Illumina genotyping chip and variant calling

Genotyping of SCAD, FMD, and MGI samples were conducted by the University of Michigan DNA Sequencing Core using the Illumina Infinium HTS Assay Protocol, a semi-custom Infinium CoreExome-24v1.1 BeadArray with 607,778 SNP markers (UM_HUNT_Biobank_v1-1_20006200_A), and the Illumina GenomeStudio v2011.1. This GWAS+exome chip platform includes standard genome-wide tagging SNPs (*N* ~ 240,000), exomic variants (*n* ~ 280,000), and custom content from previously published GWASs, additional exonic variants selected from sequencing studies, ancestry informative variants, and Neanderthal variants. Data Analysis Software package with Genotyping Module v1.9.4 and Illumina GenomeStudio (version 2.0) were used to cluster and call genotypes. Sample filtering was performed to exclude samples with call rate <98%, estimated contamination >2.5% (BAF regress), chromosomal missingness >5 times other chromosomes, and sex mismatch between genotype-inferred sex and reported gender. Variant filtering was performed to exclude probes that could not be perfectly mapped to the human genome assembly (Genome Reference Consortium Human genome build 37 and revised Cambridge Reference Sequence of the human mitochondrial DNA; BLAT); Hardy–Weinberg equilibrium (HWE) deviations in European ancestry samples (*P* < 0.00001); variant call rate <98%. Basic QC filters including HWE *P* < 0.000001 and variant missing call rate >2% were implemented for each of our laboratory chip data and MGI chip data, before combining the two data sets.

We merged all genotype data from SCAD and FMD cases and then applied pre-HRC-imputation QC using the HRC Imputation preparation and checking tool by the McCarthy Group before merging them with MGI genotyped data, which applied the same pre-HRC-imputation QC. It compares each of our individual genotyped data with HRC reference and corrects the variants strand-flip as well as aligns the allele codes with HRC reference alleles. It also removes A/T and G/C SNPs if MAF > 0.4, SNPs with differing alleles, SNPs with >0.15 allele frequency difference, and SNPs not in reference panel. After this process, 351,487 polymorphic variants remained (chr1–23). The total genotyping rate was 0.99.

### Discovery study SCAD sample QC

Two hundred and seventy-seven cases with SCAD were collected by the UBC. Based on the genotyping result, we excluded one duplicate based on whole-genome genotyping data identity-by-descent (IBD) analysis, two gender mismatched samples, and two that are further confirmed not SCAD cases. We further identified genetic syndrome cases of SCAD (*n* = 2) and removed these individuals from the analysis groups. All samples have missing call rate <1%. None of them fail QC in inbreeding coefficient check. In total, 270 SCAD samples were in our final GWAS study, which included 29 men and 241 (89.30%) women. Their average age was 53.3 ± 9.7 years. Thirteen thousand seven hundred and fifty-six MGI control subjects were included in our initial database (54.8% women, average 53.1 ± 16.4 years). Two were removed due to IBD analysis that recognized them as duplicates. All samples passed gender and inbreeding coefficient check and have missing call rate <1%. For the merged data, we further confirmed that none of the SCAD cases and MGI controls were duplicates or overlapped based on the IBD analysis. In our study, we removed the duplicates but kept the related samples. Most of the sample QC were done by PLINK v1.9^59^.

### Replication study SCAD sample QC

One hundred and sixty-five cases with SCAD were collected for replication analysis. The same sample QC as discovery stage study was applied. We removed one sample that is confirmed not SCAD case and one sample that has missing call rate >2%. No genetic syndrome cases were identified. One hundred and sixty-three SCAD cases were used in the replication. The average age was 50.5 ± 10.4 years, which included 90% women. They were genotyped using the same GWAS array as the discovery cohort and imputed together with our discovery stage samples but were only examined for the top variants of discovery result. The association was tested using SAIGE with the same study design, which were age, sex, and ancestry (Locating Ancestry from SEquence Reads (LASER)/TRACE PCs) matched controls from the MGI samples, which were exclusively independent samples from previously used controls. We also confirmed that there were no overlapping samples between discovery stage and replication based on combining IBD analysis. The sample size for the SCAD replication analysis was 163 UBC SCAD cases and 3207 MGI controls (from 1 up to 21 ratio).

### Imputation to HRC

After QCs described above, we then imputed autosomal chromosome genotypes of the HRC using the Michigan Imputation Server on the 13,756 MGI and 433 UBC SCAD (discovery+replication) samples^26,60^. The parameters for imputation included: (1) *Minimac4* method, (2) HRC r1.1 2016 reference panel, (3) *Eagle v2.3* as phase output, and (4) EUR as QC population. We filtered poorly imputed variants (*R*^2^ < 0.8) and rare variants (MAF <1%). We also excluded SNPs with potential frequency mismatches comparing with reference panel (markers with Chi-squared >300). We had 6,690,240 imputed variants (chromosomes 1–22) after the filter. The correlation *r*^2^ between the reference allele frequency of our samples and the HRC reference panel was 0.999.

### Ancestry estimation by PCA

We used TRACE in LASER software v3.0.0 to compute five PCs based on the genotype data to map the individual’s genetic ancestry using world-wide HGDP samples as reference^61^. It applied PCA of the reference panel to construct *K*-dimensional reference ancestry space using *K* PCs. We observed two main clusters in our SCAD while generating the PCA plots (PC1 to PC2). One cluster was located in East Asia (*N* = 25 for discovery samples and *N* = 34 for discovery+replication samples) and the other spread through Europe to Central South and West Asia (*N* = 245 for discovery samples and *N* = 399 for discovery+replication samples). As a sensitivity analysis, we also excluded the 25 East Asian samples to run our discovery stage association analysis.

### Matching of MGI controls to SCAD cases

In our case–control matching design, to reduce the possible false positives in our GWAS result, we required controls to have the same gender, close birth years, and close ancestries. We expected that every case could be matched to at least one control. We took a greedy approach that we searched from +/−5 years (5-year window) in age first, followed by +/−10 years instead, and so on to +/−30 years. We stopped searching once there was at least one control selected. From the possible controls in the applicable sex and age category, we chose the best ethnic match for each case that had the smallest PC distance (via the top three PCs given by TRACE program in LASER server)^62,63^. Furthermore, to guarantee every case could match to at least one and the maximal number of controls, we repeated the entire procedure 21 times (this number is decided via testing back and forth) until we used up all of the available controls that were filled in our selection criteria. In our final study, we had 270 SCAD cases (53.3 ± 9.7 years old, 89.3% female) and 5263 matched controls (52.9 ± 15.3 years old, 88% female) that was used in discovery stage GWAS and 163 SCAD cases (50.5 ± 10.4 years old, 90% female) and 3207 matched controls (49.2 ± 15.5 years old, 89% female) in replication study (case–control from 1 up to 21 ratio).

### Genome-wide association analysis accounting for imbalanced case-to-control ratios

We implemented SAIGE v0.36.1^25^, which introduces a scalable and accurate generalized mixed model association test that utilizes the saddlepoint approximation to calibrate the distribution of score test statistics. SAIGE can provide more accurate *P* values even when case–control ratios are extremely unbalanced, efficiently controlling and minimizing the type I error rates due to case–control imbalance and sample relatedness in large-scale genetic association studies. In the discovery stage and replication stages, we performed association testing for the SCAD status using SAIGE program for single genetic variants, with the first five PCs as covariates. Six million six hundred and ninety thousand two hundred and forty SNPs (after filter of imputation *r*^2^ ≥ 0.8 and MAF ≥0.01) were tested in the discovery stage and the SNPs with *P* < 5 × 10^−8^ were re-examined in replication stage using SAIGE. SNPs that retained *P* < 0.05/(number of tested SNPs) in the replication analysis were reported. Manhattan plots and QQ plots were generated from genome-wide SNPs. *λ*_GC_ was evaluated for population stratification.

### Meta-analysis of SCAD discovery and replication GWAS results

Individual genome-wide SAIGE association results of discovery stage and replication stage were then taken to meta-analysis. The METAL program^64^ was executed to combine *P* values across discovery and replication studies by taking into account each study weight effect size estimates using the inverse of the corresponding standard errors. We applied genomic control correction to all input files of whole-genome data. Manhattan plots and QQ plots were inspected as well. To visualize GWAs results for the top loci, gene locus plots (index SNP +/−500 Kb) for the top loci were generated using LocusZoom tool^65^. All SNPs with *P* < 1.0 × 10^−4^ in the SCAD meta-analysis were reported.

### Identification of independent SCAD-associated loci

From the all SAIGE association results and the meta-analysis result, we used the PLINK^59^ function “clump” for *P* < 0.0001 variants by linkage disequilibrium (LD) *r*^2^ > 0.2 in a window size of +/−500 Kb to obtain the independent loci. A further conditional test was conducted using SAIGE to test the independency of two loci whose distance was <1 Mb in the same chromosome after clump. Individual conditional results in discovery studies and replication studies by the top SNP are taken to meta-analysis by METAL. After conditioned by the top SNP, the strength of the significance of the tested SNPs was examined.

### FMD sample QC and analysis of SCAD-associated loci

Clinical replication sources were based on 412 adult FMD samples. These samples underwent the same QC as we had for SCAD samples, which included excluding duplicates samples with gender mismatch and samples with missing call rate >1%. No samples failed QC due to inbreeding coefficient checks. IBD analysis was performed to confirm that no duplicates or closely related samples (IBD PI-HAT >0.35) existed. The mean age was 53.3 ± 10.9 years, and 97.8% of FMD cases were women. Samples were collected for clinical inspection for their association between the number of risk alleles in top SCAD SNPs and the status of arterial dissections including SCAD, aneurysms, and FMD. These samples were genotyped using the same GWAS array as the discovery cohort and imputed together with our SCAD GWAS samples but were only examined for the top variants identified by our SCAD analyses. Informed consent was obtained from all participants and approval was obtained from respective Institutional Review Boards.

### SCAD heritability estimation

We fitted a univariate linear mixed model (LMM) for estimating the PVE by typed genotypes (i.e., “SNP heritability”) using GEMMA, a software implementing the Genome-wide Efficient Mixed Model Association algorithm for GWASs^66^. We use the discovery SCAD GWAS whole imputation data for analysis using a univariate LMM, and SNPs with MAF >0.05 were included in the analysis. The individual-level data used in the analysis includes 5577 samples and 5,020,100 SNPs. We use restricted maximum likelihood estimate average information algorithm in GEMMA for estimating PVE.

### Transcript expression analysis of SCAD-associated loci in GTEx

Based on our SCAD-associated loci, we queried the GTEx portal^27^ to compare genes prioritized in the colocalization analysis in different tissues and differences according to sex. Major eQTL associations (FDR ≤ 0.05) were queried based on specific associated alleles, such as rs12740679 (G/C) and *ADAMTSL4* (ENSG00000143382.14), inr aorta, coronary artery, and tibial artery. Significant variant–gene associations from GTEx v7 was based on permutations with *q* value <0.05. Sex differences were determined through DESeq analysis of GTEx RNA-Seq V7 and visualized through boxplots of transcripts per million by sex. DESeq estimates variance-mean dependence in count data from high-throughput sequencing assays and tests for differential expression based on a model using the negative binomial distribution. Violin plots and corresponding *P* values of the normalized transcript expression levels for carriers with zero risk alleles for SCAD, one risk allele, and two risk alleles in for each lead SNP were obtained through the GTEx eQTL Dashboard. Associations were calculated by linear regression, methods of which are listed in the reference for GTEx V7^67^. For comparison, similar analyses were performed for additional top loci identified with *P* < 5 × 10^−8^ in the GWAS meta-analyses.

### Colocalization analysis to prioritize genes in the SCAD-associated loci

For the significant eQTLs identified by single-variant GTEx eQTL querying for our SCAD-associated SNPs, the colocalization analysis was performed as a follow-up confirmation to test whether the leading variant of the GWAS and the eQTL signal is the same. A GWAS locus that colocalized with eQTL should be one of the primary and scalable candidate signals for follow-up functional and mechanism analyses. Two tools were utilized: coloc^68^ and locuscompareR^69^ in R program, both of which compare between eQTL result and GWAS result, taking into account LD information in the targeted gene region. The eQTL data set was downloaded from GTEx Analysis V7 (dbGaP Accession phs000424.v7.p2), and the combining results across coronary, tibial, and aorta arterial tissues were retrieved for each transcript. The meta-analysis result of SCAD GWAS analysis was compared with the eQTL result for each gene. (Approximate) Bayes Factor colocalization analyses were adopted, which embedded the concept that association of each trait with SNPs in a region may be summarized by a vector of 0s and at most a single 1, with the 1 indicating the causal SNP (assuming a single causal SNP for each trait). The posterior probability of each possible structure can be calculated as well as the posterior probabilities that the traits share their structures. The function coloc.abf() in coloc was used to test the posterior probabilities for: (H0) neither trait has a genetic association in the region; (H1/H2) only one trait has a genetic association in the region; (H3) both traits are associated but with different causal variants; (H4) both traits are associated and share a single causal variant. A posterior probability of (H4) ≥75% suggests strong evidence of the eQTL–GWAS pair influencing both the expression and GWAS trait at a particular region. The locuscompareR package further helps to visualize the colocalization events, which generates a combined plot with two locus-zoom plots (eQTL and GWAS in the same gene region) and a locus-compare scatter plot (eQTL −log10(*P*) to GWAS −log10(*P*)). The figure indicates whether the GWAS top locus is also the leading SNP in the eQTL result (both traits are associated and share a single causal variant).

### Immunohistochemistry and RNA in situ hybridization to assess human coronary artery *ADAMTSL4* expression

Human coronary arteries were fixed and paraffin embedded. De-paraffinized sections were treated with target retrieval solution DAKO citrate pH 6.1 (Agilent, Santa Clara, CA). Slides were labeled with anti-ADAMTSL4 (Sigma, St. Louis, MO) on the DAKO Autostainer (Agilent, Carpinteria, CA) using ImmPRESS Rb (Vector Labs, Burlingame, CA) and diaminobenzadine as the chromogen. Slides were imaged on EVOS microscope (ThermoFisher, Waltham, MA).

In situ hybridization for *ADAMTSL4* mRNA was performed on a normal human coronary artery sample. Five-micron sections were obtained from the fixed and paraffin-embedded human coronary artery, and slides were processed according to the RNAscope Multiplex Fluorescent V2 assay manufacturer’s instructions (ACDbio, Newark, CA). The probes used were as follows: C1- Hu TAGLN, C2-Hu ADAMTSL4, C3-HU ACTA2. Opal fluorophores (520, 570, 690) were used at 1:1000 dilution (Akoya Biosciences, Marlborough, MA). Slides were imaged on Nikon Ti microscope using the Element Software (Nikon, Melville, NY).

### Analyses of pleiotropy

We selected the top independent loci with FDR *q* value < 0.05 in the SCAD GWAS meta-analysis after selecting independent loci with LD pruning of *r*^2^ > 0.2, +/−500 Kb, and demonstrating independence after the conditional analyses (*N* = 7 loci) to search for derived PheWAS association (*P* < 1.0 × 10^−4^) in the UKB database (http://geneatlas.roslin.ed.ac.uk/phewas/). The FDR analysis was conducted based on meta-analysis result (*P* values) of 324,087 genome-wide LD-pruning SNPs that have MAF >1% and after clumping of *r*^2^ > = 0.2 at +/−500 Kb window with no *P* value filter, via R package qvalue^70^.

### Polygenic risk scores

To investigate whether our top SCAD loci are predictive for features in the FMD cases, the PRS analysis was conducted by using the polygenic scores from 7 independent loci (FDR *q* value <0.05 from 324,087 LD-pruning genome-wide loci of SCAD GWAS meta-analysis and conditional test). These seven SNPs comprised the PRS_SCAD_. We only focused on FMD cases. Logistic regression was conducted to test the association of the weighted PRS (aggregated number of risk alleles weighted by beta in our SCAD GWAS) and as a sensitivity analysis the unweighted PRS (sum of SCAD-associated risk alleles), or individual SNPs, with the binary status of subtypes in dissection, aneurysm, and FMD, including age and sex as covariates.

The genotyping array analysis of DNA from UKB participants has been previously described^55^. We evaluated the PRS_SCAD_ in 373,056 individuals of British white genetic ancestry. Weighted polygenic scores were calculated as a continuous variable using the equation: Σ(*β*_1_ × SNP_1_) + … + (*β*_7_ × SNP_7_), where *β*_*x*_ denotes the beta coefficient for the association of SNP_*x*_ with risk of SCAD and SNP_*x*_ denotes the number of SCAD-associated risk alleles (0, 1, or 2).

For the MVP cohort, we tested the association between PRS_SCAD_ and CAD/MI status using logistic regression adjusted for age (at the time of event in cases and at the time of last VA visit prior to August 2018 for controls), sex, and the first 10 ethnic-specific genetic PCs.

### PRS_CAD_ evaluation in SCAD

We tested autosomal SNPs reported in the literature as associated with CAD by GWAS-Catalog^31^ reported traits of CAD (defined as MI, percutaneous transluminal coronary angioplasty, coronary artery bypass grafting, angina, or chromic ischemic heart disease). For further PRS analysis, we removed variants without risk allele or beta information reported and without data in our SCAD GWAS result. All reported SNPs were LD-pruned based on *r*^2^ > 0.2 in +/−500 Kb of index SNPs. If same variant was reported in multiple publications, we chose the ones with strongest effect estimate (beta) reported. There were totally 386 CAD-associated SNPs selected for final PRS_CAD_ analysis. We conducted PRS analysis using GTX tool package (https://www.rdocumentation.org/packages/gtx/versions/0.0.8) in R, which uses the summary statistics of our meta-analysis results of the SCAD discovery and replication GWAS results from SAIGE. The beta coefficients of the same loci were aligned to the same effect allele between published CAD association data and our SCAD GWAS data.

### LD assessment for neighboring signals in the top-ranked SCAD loci

For the SNPs with *P* < 1.0 × 10^−8^ in our top chr1q21.2–chr1q21.3 region, LD *R*-square was examined using the Phase 3 (Version 5) of 1000 Genomes Project CEU subpopulation references by the LDlink program^43,71^, which is a web-based tool to interrogate LD in population groups. Low correlation corresponded to *r*^2^ < 0.2.

### UKB Cox regression models for risk of MI

MI events were defined pre-enrollment by self-reported medical history and post-enrollment by hospital episode statistics using ICD, Version 10 diagnosis codes (I21, I22, I23, or I24). Events were censored on the date of loss to follow-up, death, or if individuals remained event free up to March 31, 2017.

Time-to-event analyses were performed with the “survival” version 2.43-3 package for R version 3.5.1 using unadjusted and adjusted Cox regression models with years of age as a timescale. Cox regression models were adjusted for genetic sex (when not stratified by genetic sex), genotyping array and batch, and the first four PCs of ancestry. Tests for interaction were assessed between genetic features and sex.

### UKB phenome-wide association study

ORs stratified by genetic sex were calculated for the association of the PRS_SCAD_ of seven top-ranked SNPs identified in the main SCAD GWAS meta-analysis that was described before with self-reported history of MI and migraine. Results were derived from logistic regression analyses adjusted for age at enrollment, genotyping array and batch, and first four PCs of genetic ancestry.

We used the PHESANT software for R version 3.5.1 to perform a phenome-wide association study of weighted and continuous PRS_SCAD_ with 2356 phenotypes related to self-reported history of cancers, non-cancer illnesses, operations, and medications assessed at study enrollment (https://github.com/MRCIEU/PHESANT)^67^. Analyses were performed using a logistic regression with the covariates of age at enrollment, genetic sex, genotyping array and batch, and first four PCs of genetic ancestry with standardization of weighted PRS_SCAD_ variable. Two-sided *P* values were considered significant when below a Bonferroni-adjusted threshold (0.05/2356 ≈ 2.12 × 10^−5^).

### Reporting summary

Further information on research design is available in the Nature Research Reporting Summary linked to this article.

## Supplementary information

Supplementary Information

Description of Additional Supplementary Data

Supplementary Data 1

Supplementary Data 2

Supplementary Data 3

Supplementary Data 4

Supplementary Data 5

Supplementary Data 6

Supplementary Data 7

Supplementary Data 8

Reporting Summary

## Data Availability

GWAS summary statistics are available through the NHGRI-EBI GWAS Catalog under accession numbers GCST90000582 and GCST90000583. The analysis results of expression QTLs is available in our Supplementary Tables and the corresponding GTEx data are available on the GTEx project portal. The GTEx data used for the analyses described in this manuscript were obtained from RNA-Seq gene read counts from GTEx Analysis V7 downloaded 4/18/19 and Single-Tissue eQTL results from the Variant Page on GTEx Portal on 10/25/19. The GTEx data used for the analyses described in this manuscript were obtained from RNA-Seq gene read counts from GTEx Analysis V7 downloaded 4/18/19 and Single-Tissue eQTL results from the Variant Page on GTEx Portal on 10/25/19.
