# Supplementary material for: Chromosome 1q21.2 and additional loci influence risk of spontaneous coronary artery dissection and myocardial infarction
Source: Nat Commun. 2020 Sep 4;11:4432. doi: 10.1038/s41467-020-17558-x (PMC7474092; doi:10.1038/s41467-020-17558-x)
Supplement: Supplementary file 1 — Supplementary Information [file 41467_2020_17558_MOESM1_ESM.docx]

**Supplementary Information**

**Chromosome 1q21.2 and additional loci influence risk of spontaneous coronary artery dissection and myocardial infarction**

Saw J, Yang M, Trinder M, et al.

**Supplementary Table 1. Clinical characteristics of the discovery SCAD samples (N=270)**

|  | **Mean (SD)** |
| --- | --- |
| Age (yrs) | 53.3 (9.7) |
| Weight (kg) | 75.1 (19.7) |
| Height (cm) | 167.1 (7.6) |
| BMI | 26.8 (6.6) |
| Total cholesterol (umol/l) | 4.5 (1.1) |
| LDL cholesterol (umol/l) | 2.5 (0.9) |
| HDL cholesterol (umol/l) | 1.5 (0.4) |
| Triglycerides (umol/l) | 1.2 (0.6) |
| C-reactive protein( mg/l) | 12.1 (26.9) |
|  | **Count (%)** |
| Female | 241 (89.3) |
| Ethnicity |  |
| European ancetsry | 236 (87.4) |
| African Canadian | 0 (0) |
| East Asian (China, Japan) | 21 (7.9) |
| First Nation | 2 (0.8) |
| South Asian (India and sub-continent) | 8 (3.0) |
| Other | 3 (1.1) |
| Smoking (ever) | 84 (31.1) |
| Age < 50 years | 102 (37.8) |
| History of autoimmune disorder | 15 (5.6) |
| History of connective tissue disorder | 6 (2.2) |
| Systemic inflammatory disorder^1^ | 15 (5.6) |
| Genetic disorder^2^ | 3 (1.1) |
| History of stroke | 8 (3.0) |
| History of diabetes | 9 (3.3) |
|  | **Count (%)** |
| Female | 241 (89.3) |
| History of hypertension | 93 (34.4) |
| History of dyslipidemia | 68 (24.8) |
| History of Myocardial infarction (MI)^3^ | 26 (9.6) |
| Migraine headache | 89 (32.9) |
| FMD | 143 (60.9) |
| Multivessel FMD | 76 (32.3) |
| Any prior arterial aneurysm | 37 (15.7) |
| Any prior arterial dissection | 13 (4.8) |
| Intracranial aneurysm | 20 (8.5) |
| Family history of FMD | 9 (4.0) |
| Family history of arterial dissection | 12 (5.4) |
| Family history of SCAD | 9 (4.0) |
| Family history of aneurysm | 40 (17.9) |
| Grand multigravida (≥5 pregnancies) | 27 (10.5) |
| Multiparous (≥4 live birth) | 21 (8.2) |
| Peripartum (3d trimester pregnancy n=1 or 1 year postpartum) | 7 (3.2) |
| Postmenopausal | 149 (62.9) |
| Recurrent SCAD | 29 (10.7) |
| Multivessel SCAD involvement | 35 (13.0) |
| Type 1 SCAD per patient rate | 95 (34.6) |
| Type 2 SCAD per patient rate | 166 (61.7) |
| Type 3 SCAD per patient rate | 30 (11.2) |

^1^ Churg-Strauss Syndrome, Crohn’s Disease, Ulcerative Colitis, Giant Cell Arteritis, Kawasaki’s disease, Celiac disease, Wegener’s

Granulomatosis, Sarcoidosis, Polyarteritis Nodosa (PAN), or chronic hepatitis

^2^ Any known heritable condition, quite general field

^3^ Any MI prior to SCAD

**Supplementary Table 2. LD pruning and conditional analyses of the SCAD GWAS meta-analysis results to assess independence of top-ranked loci.**  Loci with false discovery rate (FDR) q value <0.05 in the GWAS meta-analysis (8 loci), selecting from SNPs with r^2^<0.2 with the index SNP in each locus and within +/-500Kb of the index SNP, were evaluated by conditional analyses within each locus. *P* values here are two-sided and un-adjusted from multiple correction. The FDR analysis was based on SCAD GWAS meta-analysis result of 324,087 genome-wide LD-pruned SNPs (r^2^<0.2 by +/- 500Kb window) with MAF>1%, which yields q-value that is an analog of the *P* value that incorporates multiple testing correction. Conditional analyses were based on meta-analysis of individual conditional results of discovery studies (N=5,533) and replication studies (N=3,370). rs12040949 in 1q21.3 was conditioned by the nearby top SNP rs12740679 in 1q21.2 for testing its independence of association. After conditional analysis, 7 top SCAD loci were available for testing in the PRS_SCAD_ analyses. Results from another independent FMD cohort replication (28 un-related SCAD cases versus 355 Cleveland Clinic Genebank controls, using logistic regression) are also shown for the 7 independent loci. All of the association models here are adjusted for PCs; and age, sex, PCs-matched between cases and controls.

|  |  |  |  |  |  | **SCAD Meta-analysis (Ncases= 433, Ncontrols= 8,470)** | | | | | | | |
| --- | --- | --- | --- | --- | --- | --- | --- | --- | --- | --- | --- | --- | --- |
| **Chr** | **Position** | **rsID** | **Locus** | **Effect Allele** | **Other Allele** | **Rsq^3^** | **OR[95%CI]** | **β** | **s.e.** | ***P*** | **EAF** | **qvalue^1^** | **FDR^1^** |
| 1q21.2 | 150262270 | rs12740679 | near *C1orf51* | G | C | 0.95 | 1.80[1.54-2.11] | 0.59 | 0.08 | 2.19E-12 | 0.26 | 0.000 | 0.001 |
| 12q13.3 | 57527283 | rs11172113 | *LRP1* | T | C | 0.99^G^ | 1.51[1.31-1.73] | 0.41 | 0.07 | 2.63E-08 | 0.61 | 0.001 | 0.002 |
| 6p24.1 | 12903957 | rs9349379 | *PHACTR1* | A | G | 0.99^G^ | 1.50[1.3-1.71] | 0.40 | 0.07 | 4.36E-08 | 0.62 | 0.001 | 0.003 |
| 21q22.11 | 35593827 | rs28451064 | near *LINC00310* | G | A | 0.90 | 1.82[1.47-2.26] | 0.60 | 0.11 | 1.19E-07 | 0.87 | 0.003 | 0.013 |
| 6q25.3 | 155813320 | rs78349783^&^ | near *NOX3* | G | A | 0.95 | 1.75[1.41-2.17] | 0.56 | 0.11 | 1.03E-06 | 0.12 | 0.019 | 0.091 |
| 2q33.2 | 203911272 | rs78377252 | *NBEAL1* | A | G | 0.81 | 3.26[1.99-5.31] | 1.18 | 0.25 | 2.43E-06 | 0.02 | 0.038 | 0.091 |
| 1q21.3 | 150447462 | rs12040949* | *RPRD2* | C | T | 0.97 | 1.41[1.22-1.61] | 0.34 | 0.07 | 3.05E-06 | 0.63 | 0.038 | 0.091 |
| 1p32.1 | 59635971 | rs11207415^&^ | near *HSD52* | T | C | 0.97 | 1.46[1.25-1.71] | 0.38 | 0.08 | 3.23E-06 | 0.27 | 0.038 | 0.091 |
|  |  |  |  |  |  |  |  |  |  |  |  |  |  |

|  |  |  |  |  |  |  |  |  |  |  |  |  |  |
| --- | --- | --- | --- | --- | --- | --- | --- | --- | --- | --- | --- | --- | --- |
|  |  |  |  |  |  | **FMD cohort replication in independent samples (28 cases v.s. 355 controls)^2^** | | | | | | | |
| **Chr** | **Position** | **rsID** | **Locus** | **Effect Allele** | **Other Allele** | **Rsq^3^** | **OR[95%CI]** | **β** | **s.e.** | ***P*** | **AF.Cases** | **AF.Controls** | **EAF** |
| 1q21.2 | 150262270 | rs12740679 | near *C1orf51* | G | C | 0.96 | 1.92[1.06-3.45] | 0.65 | 0.30 | 0.032 | 0.38 | 0.24 | 0.25 |
| 12q13.3 | 57527283 | rs11172113 | *LRP1* | T | C | 1.00^G^ | 1.77[0.96-3.25] | 0.57 | 0.31 | 0.068 | 0.71 | 0.59 | 0.60 |
| 6p24.1 | 12903957 | rs9349379 | *PHACTR1* | A | G | 1.00^G^ | 2.33[1.23-4.47] | 0.85 | 0.33 | 0.010 | 0.79 | 0.61 | 0.62 |
| 21q22.11 | 35593827 | rs28451064 | near *LINC00310* | G | A | 0.90 | 1.09[0.46-2.57] | 0.08 | 0.44 | 0.847 | 0.88 | 0.87 | 0.87 |
| 6q25.3 | 155813320 | rs78349783^&^ | near *NOX3* | G | A | 0.96 | 0.78[0.33-1.84] | -0.25 | 0.44 | 0.571 | 0.11 | 0.15 | 0.14 |
| 2q33.2 | 203911272 | rs78377252 | *NBEAL1* | A | G | 0.80 | 1.32[0.29-6.1] | 0.28 | 0.78 | 0.722 | 0.04 | 0.03 | 0.03 |
| 1q21.3 | 150447462 | rs12040949* | *RPRD2* | C | T | - | - | - | - | - | - | - | - |
| 1p32.1 | 59635971 | rs11207415^&^ | near *HSD52* | T | C | 0.99 | 2.18[1.24-3.85] | 0.78 | 0.29 | 0.007 | 0.43 | 0.26 | 0.27 |

^*^ rs12040949 has LD r2=0.19 with rs12740679 (from 1000G CEU population). After conditional by rs12740679, this SNP has P=0.1122 (β=0.14, s.e.=0.09) in meta-analysis. Therefore, we will remove this from our top independent SCAD loci.

^1.^ The FDR analysis was based on SCAD GWAS meta-analysis result of 324,087 genome-wide LD-pruned SNPs (filter out r2>=0.2 with index SNPs at a +/- 500Kb window) with MAF>1%. Independent loci with FDR q-value<0.05 are reported.

^2.^ Corresponding genome-wide association analysis lambdaGC =0.996

^3.^ Imputation R-square

^&^ LD r2=0.0048 between rs78349783 and rs9349379. LD r2=0.0024 between rs11207415 and rs12740679. LD was estimated by 1000G CEU population.

^G^ Genotyped SNPs

**Supplementary Table 3. SCAD- associated loci results in the discovery stage SCAD GWAS for effects with/without FMD cases in the subgroup analyses.** GWAS was based on generalized mixed models in SAIGE, which uses the saddlepoint approximation (SPA) correction that accounts for case and control imbalances. GC correction was applied. SNPs with imputation Rsq ≥0.8 and MAF ≥ 1% were analyzed. *P* values here are two-sided and un-adjusted from multiple correction. Variants with association P < 5x10-8 pass the genome-wide significance Bonferroni corrected threshold. All of the association models are adjusted for PCs; and age, sex, PC-matched between cases and controls. EAF=effect allele frequency.

|  |  |  |  |  |  | **All SCAD (N=5,533)** | | | | | | | |
| --- | --- | --- | --- | --- | --- | --- | --- | --- | --- | --- | --- | --- | --- |
| **Chr** | **Position** | **rsID** | **Rsq*** | **Effect Allele** | **Other Allele** | **OR[95%CI]** | **β** | **s.e.** | ***P*** | **EAF** | **AF Cases** | **AF Controls** |  |
| 1q21.2 | 150262270 | rs12740679 | 0.95 | G | C | 1.97[1.59-2.45] | 0.68 | 0.11 | 2.88E-10 | 0.26 | 0.39 | 0.25 |  |
| 6p24.1 | 12903957 | rs9349379 | 0.99^&^ | A | G | 1.54[1.29-1.83] | 0.43 | 0.09 | 4.14E-06 | 0.61 | 0.68 | 0.61 |  |
| 12q13.3 | 57527283 | rs11172113 | 0.99^&^ | T | C | 1.52[1.28-1.82] | 0.42 | 0.09 | 1.05E-05 | 0.61 | 0.72 | 0.60 |  |
| 21q22.11 | 35593827 | rs28451064 | 0.90 | G | A | 1.71[1.28-2.30] | 0.54 | 0.15 | 2.10E-04 | 0.87 | 0.93 | 0.87 |  |
|  |  |  |  |  |  | **SCAD + FMD (N=2,922)^a^** | | | | | | | |
| **Chr** | **Position** | **rsID** | **Rsq*** | **Effect Allele** | **Other Allele** | **OR[95%CI]** | **β** | **s.e.** | ***P*** | **EAF** | **AF Cases** | **AF Controls** |  |
| 1q21.2 | 150262270 | rs12740679 | 0.95 | G | C | 2.14[1.61-2.84] | 0.759 | 0.145 | 1.81E-07 | 0.257 | 0.41 | 0.25 |  |
| 6p24.1 | 12903957 | rs9349379 | 0.99^&^ | A | G | 1.62[1.26-2.08] | 0.482 | 0.127 | 1.45E-04 | 0.623 | 0.69 | 0.62 |  |
| 12q13.3 | 57527283 | rs11172113 | 0.99^&^ | T | C | 1.63[1.27-2.11] | 0.491 | 0.130 | 1.57E-04 | 0.601 | 0.73 | 0.59 |  |
| 21q22.11 | 35593827 | rs28451064 | 0.90 | G | A | 1.64[1.11-2.41] | 0.493 | 0.198 | 1.28E-02 | 0.876 | 0.93 | 0.87 |  |
|  |  |  |  |  |  | **SCAD without FMD (N=2,655)^b^** | | | | | | | |
| **Chr** | **Position** | **rsID** | **Rsq*** | **Effect Allele** | **Other Allele** | **OR[95%CI]** | **β** | **s.e.** | ***P*** | **EAF** | **AF Cases** | **AF Controls** |  |
| 1q21.2 | 150262270 | rs12740679 | 0.95 | G | C | 1.83[1.35-2.47] | 0.603 | 0.153 | 8.25E-05 | 0.258 | 0.37 | 0.25 |  |
| 6p24.1 | 12903957 | rs9349379 | 0.99^&^ | A | G | 1.45[1.11-1.88] | 0.370 | 0.134 | 5.90E-03 | 0.604 | 0.66 | 0.60 |  |
| 12q13.3 | 57527283 | rs11172113 | 0.99^&^ | T | C | 1.42[1.09-1.86] | 0.354 | 0.135 | 8.93E-03 | 0.614 | 0.70 | 0.61 |  |
| 21q22.11 | 35593827 | rs28451064 | 0.90 | G | A | 1.85[1.24-2.75] | 0.614 | 0.203 | 2.54E-03 | 0.873 | 0.94 | 0.87 |  |

^a^ 144 cases versus 2,778 MGI matched ctrls, λGC=0.97 *Imputation R-square

^b^ 128 cases versus 2,527 MGI matched ctrls, λGC=0.97 ^&^Genotyped SNPs

**Supplementary Table 4. Overall review of associations with the chromosome 1q21.2 locus (rs12740679) in the discovery stage primary and secondary analyses.** GWAS was based on generalized mixed models in SAIGE, which uses the saddlepoint approximation (SPA) correction that accounts for case and control imbalances. GC correction was applied. SNPs with imputation Rsq ≥0.8 and MAF ≥ 1% were analyzed. *P* values here are two-sided and un-adjusted from multiple correction. Variants with association *P* < 5x10^-8^ pass the genome-wide significance Bonferroni corrected threshold. All of the association models are adjusted for PCs, with age, sex, PC-matched cases and controls.

| **Analysis** | **Allele** | **MAF** | **N** | **β** | **s.e.** | ***P*** |
| --- | --- | --- | --- | --- | --- | --- |
| Main GWAS (all samples) | G | 0.26 | 5,533 | 0.68 | 0.11 | 2.88E-10 |
|  |  |  |  |  |  |  |
| GWAS after removing Asian SCAD cases and corresponding matched MGI controls | G | 0.26 | 5,076 | 0.66 | 0.11 | 2.17E-09 |
| Female only SCAD GWAS | G | 0.26 | 4,895 | 0.73 | 0.11 | 2.78E-10 |
| Male only SCAD GWAS | G | 0.27 | 638 | 0.34 | 0.32 | 2.76E-01 |
| SCAD without FMD GWAS | G | 0.26 | 2,655 | 0.60 | 0.15 | 8.25E-05 |
| SCAD+FMD GWAS | G | 0.26 | 2,922 | 0.76 | 0.15 | 1.81E-07 |

**Supplementary Table 5. PRS_SCAD_ "vascular PheWAS" using logistic regression models in the FMD cohort.** The PRS_SCAD_ was tested for associated (adjusted for age and sex) with dissection, aneurysm, and multifocal stenosis FMD in unrelated FMD cases (N=412) that were independent of the SCAD GWAS samples. *P* values listed are unadjusted from multiple correction (two-sided).

|  | **N=412** | | **Weighted PRS_SCAD_ score^§^** | | | | **Unweighted PRS score** | | | |
| --- | --- | --- | --- | --- | --- | --- | --- | --- | --- | --- |
| **Phenotype*** | **N.ctrls** | **N.cases** | **OR[95%CI]** | **β** | **s.e.** | **Pr(>\|z\|)** | **OR[95%CI]** | **β** | **s.e.** | **Pr(>\|z\|)** |
| HTN | 214 | 198 | 0.74[0.57-0.97] | -0.295 | 0.135 | 2.88E-02 | 0.85[0.75-0.97] | -0.159 | 0.067 | 1.71E-02 |
| ***Dissection*** |  |  |  |  |  |  |  |  |  |  |
| Any Dissection | 281 | 131 | 1.18[0.89-1.57] | 0.164 | 0.146 | 2.61E-01 | 1.11[0.96-1.28] | 0.104 | 0.072 | 1.49E-01 |
| Aorta | 409 | 3 | 0.63[0.14-2.93] | -0.462 | 0.784 | 5.55E-01 | 0.81[0.38-1.71] | -0.210 | 0.382 | 5.83E-01 |
| Cerebral | 410 | 2 | 0.82[0.13-5.37] | -0.197 | 0.958 | 8.37E-01 | 1.05[0.42-2.60] | 0.045 | 0.465 | 9.24E-01 |
| Cervical | 316 | 96 | 1.08[0.79-1.48] | 0.079 | 0.158 | 6.16E-01 | 1.06[0.91-1.24] | 0.059 | 0.078 | 4.56E-01 |
| Internal Carotid Artery | 328 | 84 | 1.05[0.76-1.45] | 0.048 | 0.166 | 7.70E-01 | 1.04[0.88-1.22] | 0.037 | 0.082 | 6.51E-01 |
| Vertebral artery | 384 | 28 | 1.61[0.97-2.68] | 0.479 | 0.259 | 6.50E-02 | 1.31[1.02-1.70] | 0.274 | 0.132 | 3.84E-02 |
| Coronary | 384 | 28 | 1.82[1.09-3.02] | 0.597 | 0.259 | 2.13E-02 | 1.40[1.08-1.82] | 0.339 | 0.133 | 1.10E-02 |
| Lower Extremity | 404 | 8 | 1.83[0.75-4.46] | 0.602 | 0.456 | 1.87E-01 | 1.22[0.77-1.93] | 0.198 | 0.235 | 3.99E-01 |
| Upper Extremity | 412 | 0 |  | - | - | - |  | - | - | - |
| Visceral | 401 | 11 | 1.35[0.57-3.19] | 0.302 | 0.438 | 4.91E-01 | 1.13[0.73-1.74] | 0.120 | 0.222 | 5.89E-01 |
| Mesenteric | 406 | 6 | 1.37[0.48-3.90] | 0.313 | 0.535 | 5.59E-01 | 1.04[0.61-1.76] | 0.035 | 0.271 | 8.96E-01 |
| Renal | 406 | 6 | 1.78[0.50-6.28] | 0.577 | 0.643 | 3.70E-01 | 1.25[0.65-2.42] | 0.226 | 0.335 | 5.00E-01 |
| ***Aneurysm*** |  |  |  |  |  |  |  |  |  |  |
| Any Aneurysm | 319 | 93 | 0.95[0.70-1.29] | -0.054 | 0.158 | 7.35E-01 | 0.97[0.84-1.14] | -0.026 | 0.078 | 7.37E-01 |
| Aorta | 401 | 11 | 1.09[0.49-2.41] | 0.086 | 0.404 | 8.32E-01 | 1.06[0.72-1.57] | 0.062 | 0.200 | 7.55E-01 |
| Cerebral | 380 | 32 | 0.67[0.41-1.11] | -0.397 | 0.254 | 1.18E-01 | 0.84[0.66-1.07] | -0.177 | 0.124 | 1.52E-01 |
| Cervical | 382 | 30 | 0.72[0.43-1.20] | -0.330 | 0.260 | 2.04E-01 | 0.88[0.69-1.13] | -0.126 | 0.126 | 3.18E-01 |
| Internal Carotid Artery | 384 | 28 | 0.68[0.40-1.16] | -0.384 | 0.270 | 1.55E-01 | 0.86[0.66-1.11] | -0.156 | 0.131 | 2.33E-01 |
| Vertebral artery | 411 | 1 | 1.06[0.05-22.13] | 0.055 | 1.552 | 9.72E-01 | 1.23[0.26-5.71] | 0.205 | 0.784 | 7.94E-01 |
| Coronary | 412 | 0 |  | - | - | - |  | - | - | - |
| Lower Extremity | 410 | 2 | 1.99[0.34-11.67] | 0.687 | 0.903 | 4.47E-01 | 1.04[0.42-2.59] | 0.040 | 0.466 | 9.32E-01 |
| Upper Extremity | 412 | 0 |  | - | - | - |  | - | - | - |
| Visceral | 372 | 40 | 1.14[0.73-1.75] | 0.127 | 0.222 | 5.66E-01 | 1.03[0.83-1.28] | 0.032 | 0.110 | 7.68E-01 |
| Mesenteric | 399 | 13 | 0.97[0.47-2.03] | -0.027 | 0.375 | 9.42E-01 | 0.90[0.62-1.29] | -0.111 | 0.186 | 5.51E-01 |
| Renal | 379 | 33 | 1.12[0.69-1.79] | 0.110 | 0.242 | 6.48E-01 | 1.06[0.84-1.34] | 0.061 | 0.120 | 6.10E-01 |
| ***FMD (arterial stenosis)*** |  |  |  |  |  |  |  |  |  |  |
| Any Multifocal FMD | 0 | 412 | 1.00 | 0.000 | 23502.153 | 1.00 | 1.00 | 0.000 | 11610.538 | 1.00 |
| Cerebral | 358 | 54 | 0.93[0.63-1.36] | -0.075 | 0.196 | 7.01E-01 | 0.96[0.79-1.16] | -0.044 | 0.097 | 6.46E-01 |
| Cervical | 86 | 326 | 1.58[1.13-2.22] | 0.460 | 0.173 | 7.89E-03 | 1.27[1.07-1.50] | 0.236 | 0.085 | 5.54E-03 |
| Internal Carotid Artery | 91 | 321 | 1.50[1.08-2.09] | 0.409 | 0.168 | 1.49E-02 | 1.23[1.05-1.44] | 0.206 | 0.082 | 1.24E-02 |
| Vertebral artery | 390 | 22 | 1.10[0.62-1.95] | 0.097 | 0.291 | 7.40E-01 | 1.09[0.82-1.45] | 0.087 | 0.144 | 5.44E-01 |
| Lower Extremity | 348 | 64 | 0.95[0.66-1.35] | -0.056 | 0.183 | 7.59E-01 | 0.99[0.83-1.18] | -0.009 | 0.090 | 9.17E-01 |
| Upper Extremity | 402 | 10 | 0.80[0.34-1.86] | -0.222 | 0.430 | 6.05E-01 | 0.89[0.59-1.35] | -0.113 | 0.211 | 5.93E-01 |
| Visceral | 159 | 253 | 0.79[0.60-1.03] | -0.239 | 0.137 | 7.99E-02 | 0.89[0.78-1.02] | -0.114 | 0.068 | 9.23E-02 |
| Mesenteric | 378 | 34 | 0.79[0.49-1.27] | -0.239 | 0.244 | 3.27E-01 | 0.92[0.73-1.16] | -0.086 | 0.119 | 4.69E-01 |
| Renal | 163 | 249 | 0.80[0.61-1.04] | -0.224 | 0.136 | 9.98E-02 | 0.90[0.79-1.03] | -0.106 | 0.067 | 1.13E-01 |

^*^ All models were adjusted for age and sex.

^§^ The PRS was weighted according to the SCAD GWAS meta-analysis beta coefficient.

**Supplementary Table 6. PRS summary and sensitivity analysis after removing the chromosome 6p24.1 PHACTR1 locus.** Results are shown for the logistic regression of PRSCAD association testing with SCAD, logistic regression PRS_SCAD_ with CAD or MI association in MVP cohort, PRS_SCAD_ with MI association in UKB by Cox proportional hazards regression models, and logistic regression PRS_SCAD_ with SCAD in the FMD cohort. *P* values here are two-sided and un-adjusted from multiple correction. All models are adjusted by age, sex, and PCs.

| **PRS (weighted scores)** | **Phenotype** | **Cohort-all samples** | **Total N** | **N.cases** | **N.controls** | **β** | **s.e.** | ***P*** | **OR[95%CI]** |
| --- | --- | --- | --- | --- | --- | --- | --- | --- | --- |
| 386 CAD SNPs | SCAD | SCAD GWAS  meta-analysis | 8,903 | 433 | 8,470 | -0.250 | 0.069 | 3.23E-04 | 0.78[0.68-0.89] |
| 385 CAD SNPs* | SCAD | SCAD GWAS  meta-analysis | 8,903 | 433 | 8,470 | -0.203 | 0.070 | 3.75E-03 | 0.82[0.71-0.94] |
| 7 SCAD SNPs | SCAD | FMD | 412 | 28 | 384 | 0.597 | 0.259 | 2.13E-02 | 1.82[1.09-3.02] |
| 6 SCAD SNPs* | SCAD | FMD | 412 | 28 | 384 | 0.551 | 0.273 | 4.34E-02 | 1.73[1.02-2.96] |
| 7 SCAD SNPs | MI | UKB | 373,056 | 15,476 | 357,580 | -0.094 | 0.011 | 1.28E-17 | 0.91[0.89-0.93] |
| 6 SCAD SNPs* | MI | UKB | 373,056 | 15,476 | 357,580 | -0.063 | 0.012 | 9.53E-08 | 0.94[0.92-0.96] |
| 7 SCAD SNPs | CAD | MVP | 294,465 | 95,347 | 199,118 | -0.050 | 0.004 | 9.33E-36 | 0.95[0.94-0.96] |
| 6 SCAD SNPs* | CAD | MVP | 294,465 | 95,347 | 199,118 | -0.031 | 0.004 | 1.39E-14 | 0.97[0.96-0.98] |
| 7 SCAD SNPs | MI | MVP | 314,434 | 14,802 | 299,632 | -0.040 | 0.009 | 3.35E-06 | 0.96[0.95-0.98] |
| 6 SCAD SNPs* | MI | MVP | 314,434 | 14,802 | 299,632 | -0.020 | 0.009 | 1.91E-02 | 0.98[0.96-1.00] |

***** removing rs9349379

**Supplementary Table 7.** PRS_SCAD_ analyzed in UKB using Cox proportional hazards regression models for MI, stratified by sex.  Models were adjusted for genetic sex (when not stratified by genetic sex), genotyping array and batch, and the first 4 principal components of ancestry. P values here are two-sided and un-adjusted from multiple correction. Age was used as the time scale.

|  | **Model for percentile of summed SCAD risk alleles** | | | | | | **Model for percentile of the weighted PRS** | | | | | |
| --- | --- | --- | --- | --- | --- | --- | --- | --- | --- | --- | --- | --- |
| **Sample** | **β** | **s.e.** | ***P*** | **HR[95%CI]^£^** | **Total N** | **#MI events** | **β** | **s.e.** | ***P*** | **HR[95%CI] ^£^** | **Total N** | **#MI events** |
| ALL^1^ | -0.048 | 0.005 | 8.00E-22 | 0.95[0.94-0.96] | 373,056 | 15,476 | -0.094 | 0.011 | 1.28E-17 | 0.91[0.89-0.93] | 373,056 | 15,476 |
| Male^2^ | -0.047 | 0.006 | 2.47E-14 | 0.95[0.94-0.97] | 171,082 | 11,751 | -0.092 | 0.013 | 1.57E-13 | 0.91[0.89-0.93] | 171,082 | 11,751 |
| Female^2^ | -0.052 | 0.011 | 2.40E-06 | 0.95[0.93-0.97] | 201,974 | 3,725 | -0.099 | 0.022 | 9.46E-06 | 0.91[0.87-0.95] | 201,974 | 3,725 |
| ALL^&1^ | -0.031 | 0.006 | 5.20E-07 | 0.97[0.96-0.98] | 373,056 | 15,476 | -0.063 | 0.012 | 9.53E-08 | 0.94[0.92-0.96] | 373,056 | 15,476 |
| Male^&2^ | -0.027 | 0.007 | 1.22E-04 | 0.97[0.96-0.99] | 171082 | 11751 | -0.058 | 0.013 | 1.93E-05 | 0.94[0.92-0.97] | 171,082 | 11,751 |
| Female^&2^ | -0.043 | 0.012 | 5.43E-04 | 0.96[0.93-0.98] | 201974 | 3725 | -0.080 | 0.024 | 8.59E-04 | 0.92[0.88-0.97] | 201,974 | 3,725 |

^&^excluding rs9349379

^£^HR= Hazard ratio= exponential of beta coefficient.

^1^adjusted genetic sex, genotyping array and batch, 4 PCs genetic ancestry

^2^adjusted genotyping array and batch, 4 PCs genetic ancestry

**Supplementary Table 8.** PRS_SCAD_ association with CAD and MI in the MVP cohort. Logistic regression P values here are two-sided and un-adjusted from multiple correction. All models are adjusted by age,sex, and PCs. Sensitivity analysis after removing the chromosome 6p24.1 PHACTR1 locus was also performed.

| **PRS-SCAD** | **Phenotype** | **Ncases** | **Ncontrols** | **Sex** | **β** | **s.e.** | **OR[95% CI]** | ***P*** |
| --- | --- | --- | --- | --- | --- | --- | --- | --- |
| weighted | CAD | 92,971 | 178,758 | M | -0.050 | 0.004 | 0.95[0.94-0.96] | 1.12E-34 |
| weighted* | CAD | 92,971 | 178,758 | M | -0.031 | 0.004 | 0.97[0.96-0.98] | 6.87E-14 |
| unweighted | CAD | 92,971 | 178,758 | M | -0.054 | 0.004 | 0.95[0.94-0.96] | 4.10E-39 |
| unweighted* | CAD | 92,971 | 178,758 | M | -0.031 | 0.004 | 0.97[0.96-0.98] | 4.86E-14 |
| weighted | CAD | 2,180 | 18,529 | F | -0.044 | 0.023 | 0.96[0.91-1.00] | 5.35E-02 |
| weighted* | CAD | 2,180 | 18,529 | F | -0.036 | 0.023 | 0.96[0.92-1.01] | 1.17E-01 |
| unweighted | CAD | 2,180 | 18,529 | F | -0.046 | 0.023 | 0.96[0.91-1.00] | 4.62E-02 |
| unweighted* | CAD | 2,180 | 18,529 | F | -0.036 | 0.023 | 0.96[0.92-1.01] | 1.10E-01 |
| weighted | CAD | 95,347 | 199,118 | all | -0.050 | 0.004 | 0.95[0.94-0.96] | 9.33E-36 |
| weighted* | CAD | 95,347 | 199,118 | all | -0.031 | 0.004 | 0.97[0.96-0.98] | 1.39E-14 |
| unweighted | CAD | 95,347 | 199,118 | all | -0.054 | 0.004 | 0.95[0.94-0.96] | 3.16E-40 |
| unweighted* | CAD | 95,347 | 199,118 | all | -0.031 | 0.004 | 0.97[0.96-0.98] | 9.73E-15 |
| weighted | MI | 14,411 | 276,025 | M | -0.039 | 0.009 | 0.96[0.95-0.98] | 6.43E-06 |
| weighted* | MI | 14,411 | 276,025 | M | -0.019 | 0.009 | 0.98[0.96-1.00] | 3.08E-02 |
| unweighted | MI | 14,411 | 276,025 | M | -0.045 | 0.009 | 0.96[0.94-0.97] | 2.30E-07 |
| unweighted* | MI | 14,411 | 276,025 | M | -0.021 | 0.009 | 0.98[0.96-1.00] | 1.43E-02 |
| weighted | MI | 367 | 21,544 | F | -0.057 | 0.053 | 0.94[0.85-1.05] | 2.81E-01 |
| weighted* | MI | 367 | 21,544 | F | -0.063 | 0.053 | 0.94[0.85-1.04] | 2.38E-01 |
| unweighted | MI | 367 | 21,544 | F | -0.047 | 0.053 | 0.95[0.86-1.06] | 3.72E-01 |
| unweighted* | MI | 367 | 21,544 | F | -0.054 | 0.053 | 0.95[0.85-1.05] | 3.08E-01 |
| weighted | MI | 14,802 | 299,632 | all | -0.040 | 0.009 | 0.96[0.95-0.98] | 3.35E-06 |
| weighted* | MI | 14,802 | 299,632 | all | -0.020 | 0.009 | 0.98[0.96-1.00] | 1.91E-02 |
| unweighted | MI | 14,802 | 299,632 | all | -0.045 | 0.009 | 0.96[0.94-0.97] | 1.37E-07 |
| unweighted* | MI | 14,802 | 299,632 | all | -0.022 | 0.009 | 0.98[0.96-0.99] | 9.27E-03 |

*excluding rs9349379

**Supplementary Figure 1. Illustration of SCAD**

**
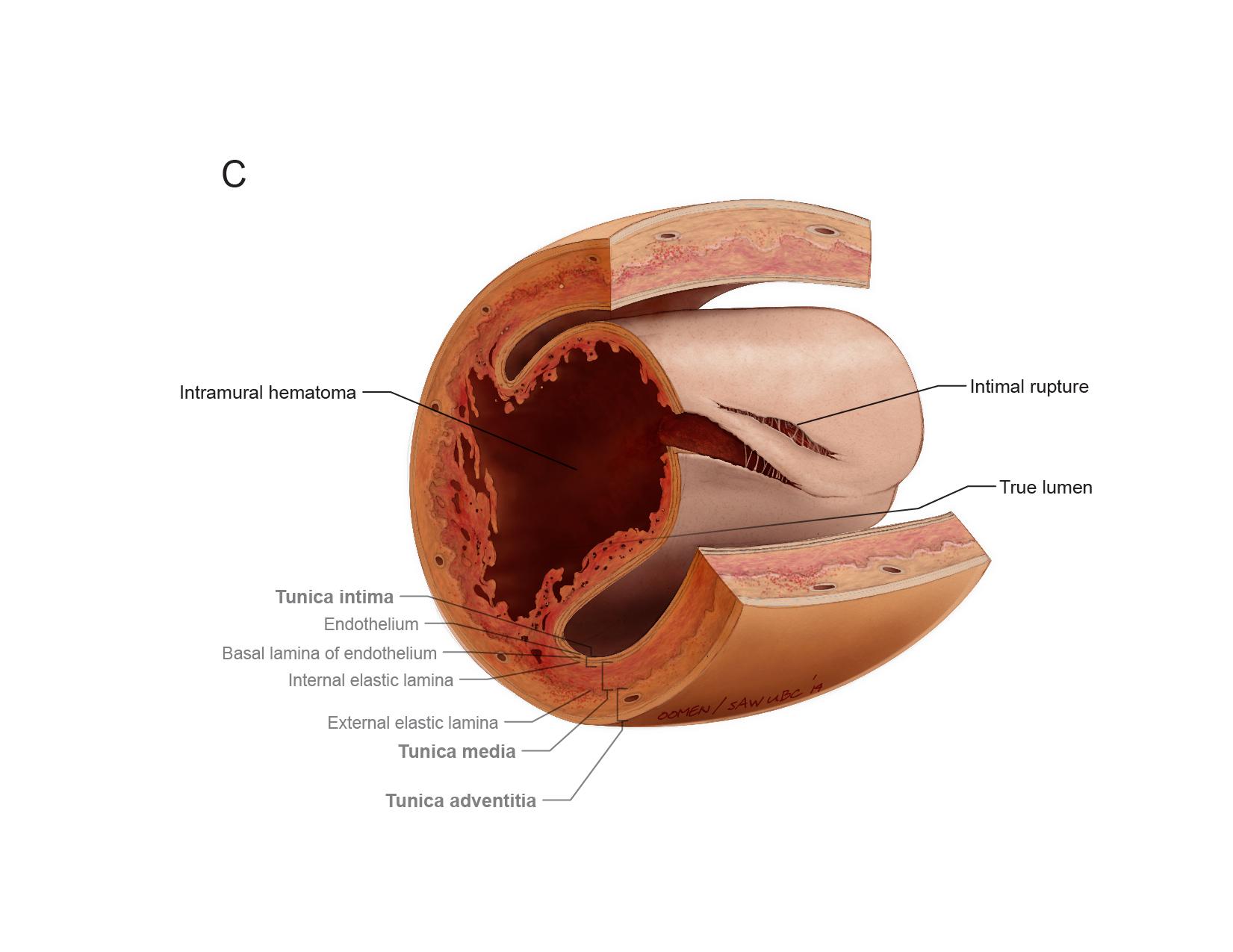
**

**Supplementary Figure 2. Ancestry estimation of SCAD samples.** **a,** Principal component analysis (PCA) plots were generated by the LASER/TRACE program of CanSCAD discovery stage participants (N=270) and world-wide human genome diversity project (HGDP) samples as the reference shows that most individuals in the SCAD discovery cohort are of European ancestry (N=245) and only a small subset of mixed or mostly East Asian ancestry in the gray circle (N=25). Black dots are CanSCAD participants and HGDP references are in different colors for different ethnicities, as indicated. **b,** PCA plots are shown separately for the SCAD discovery and replication cohorts, along with the matched MGI controls included in each GWAS.

a.


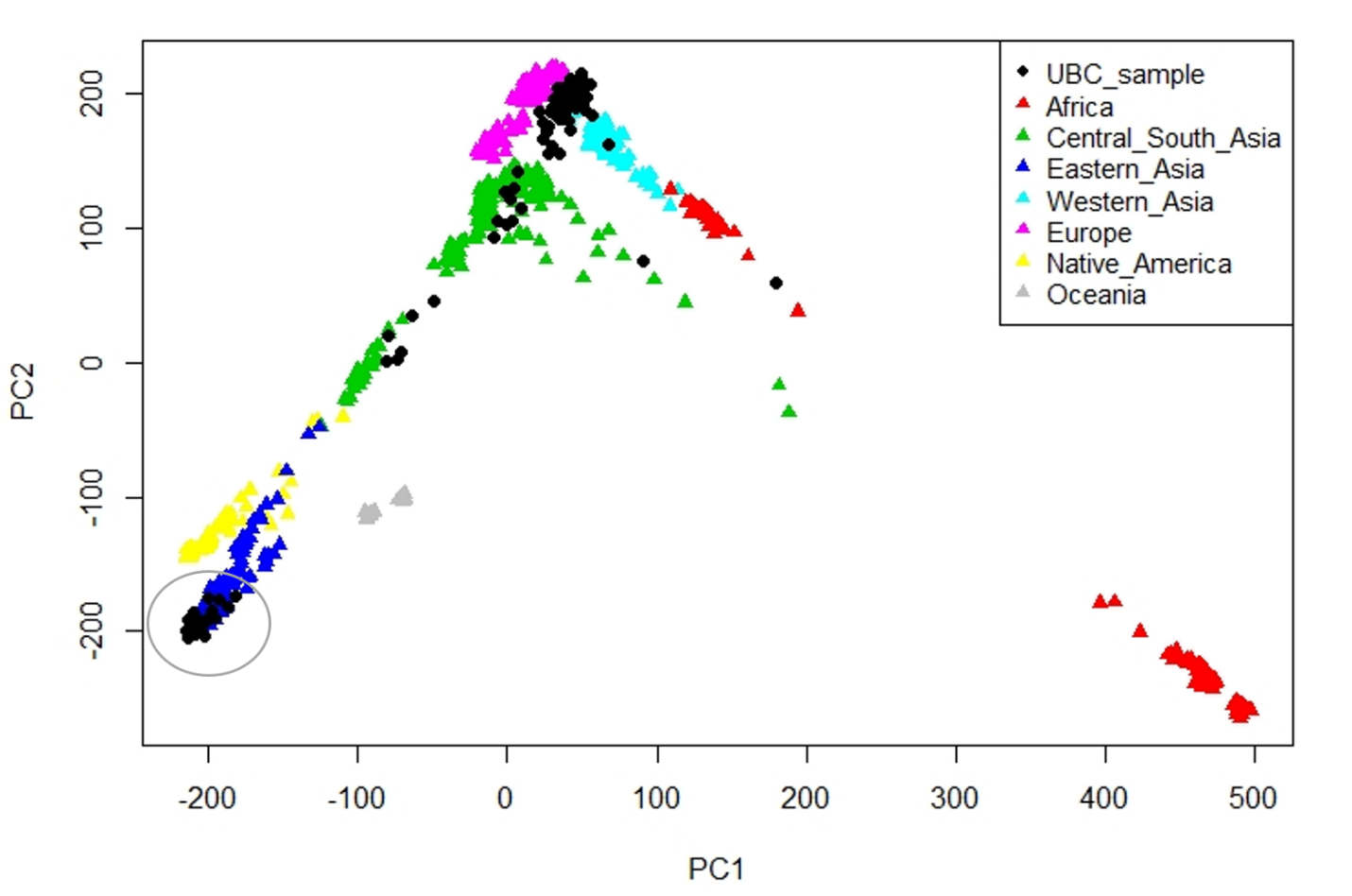


b.


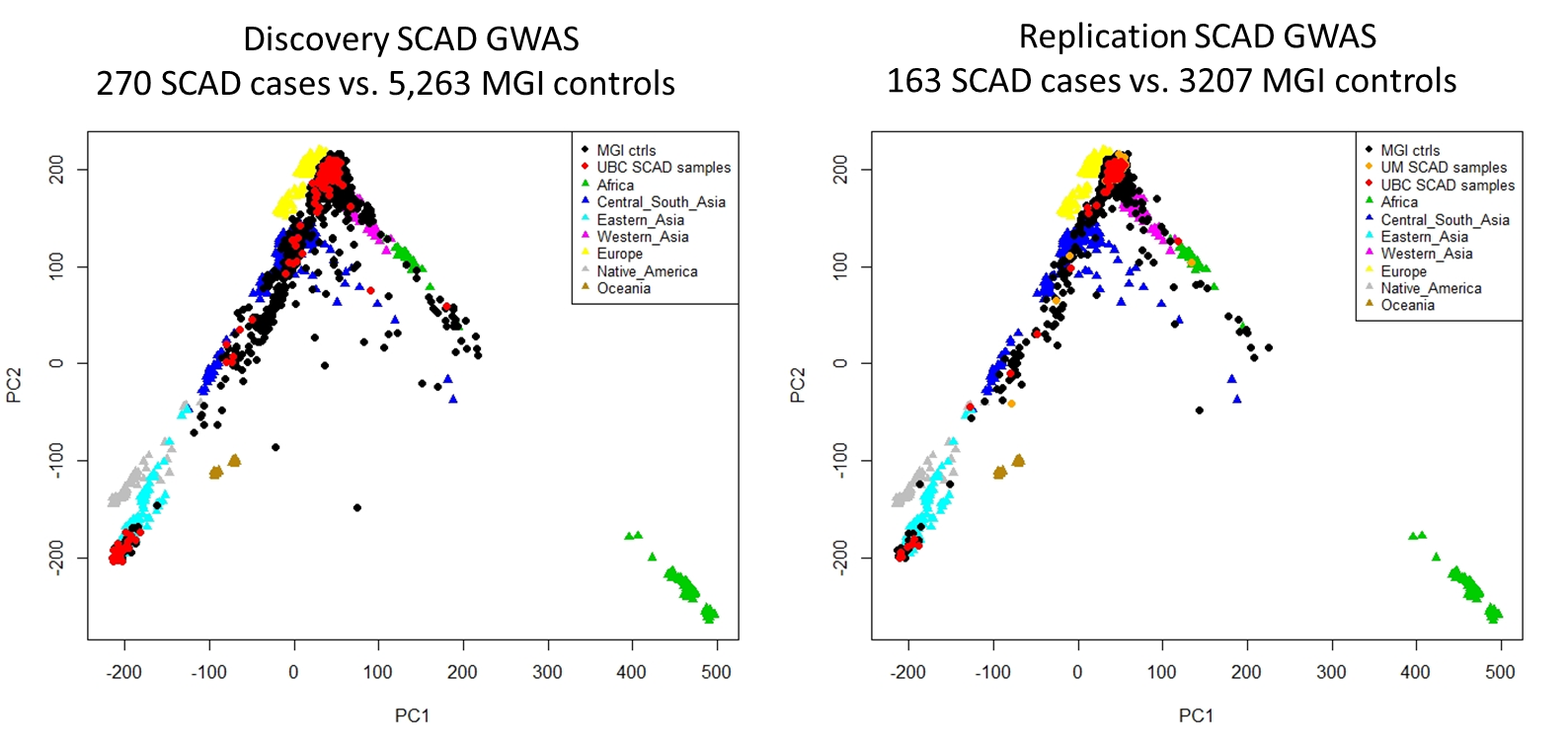

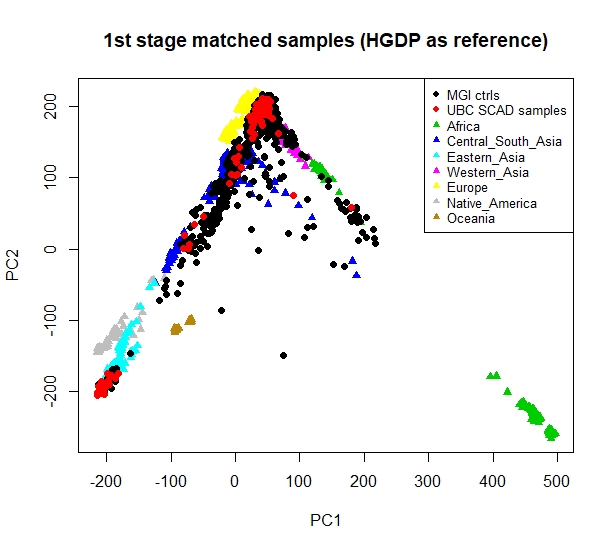

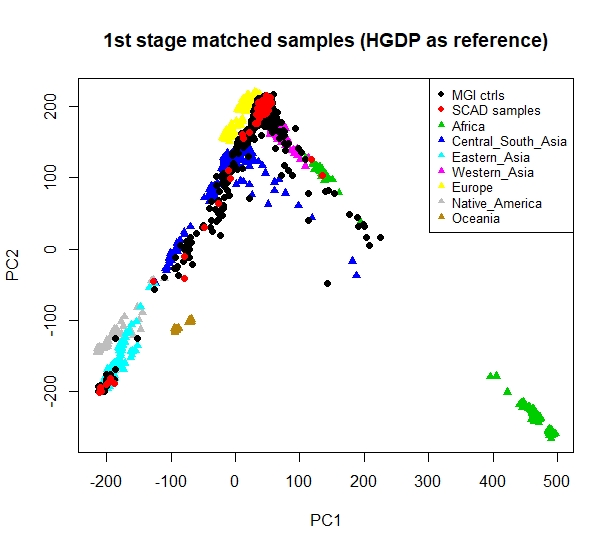


**Supplementary Figure 3. Females–only SCAD GWAS meta-analysis results.** Manhattan plots and QQ plot for the meta-analysis of female SCAD discovery and replication samples (Ncases=387, Ncontrols=7,504). Discovery GWAS and replication association analysis were all based on generalized mixed models in SAIGE, which uses the saddlepoint approximation (SPA) correction that accounts for case and control imbalances. GC correction was applied before standard error weighted meta-analysis. *P* values here are two-sided and un-adjusted from multiple correction. All of the association models are adjusted for PCs; and age, sex, PC-matched between cases and controls. SNPs with MAF ≥ 1% were analyzed, and variants meeting the genome-wide Bonferroni corrected significance threshold (*P* < 5x10^-8^) are shown in blue. The λ_GC_ value is 0.96.

**
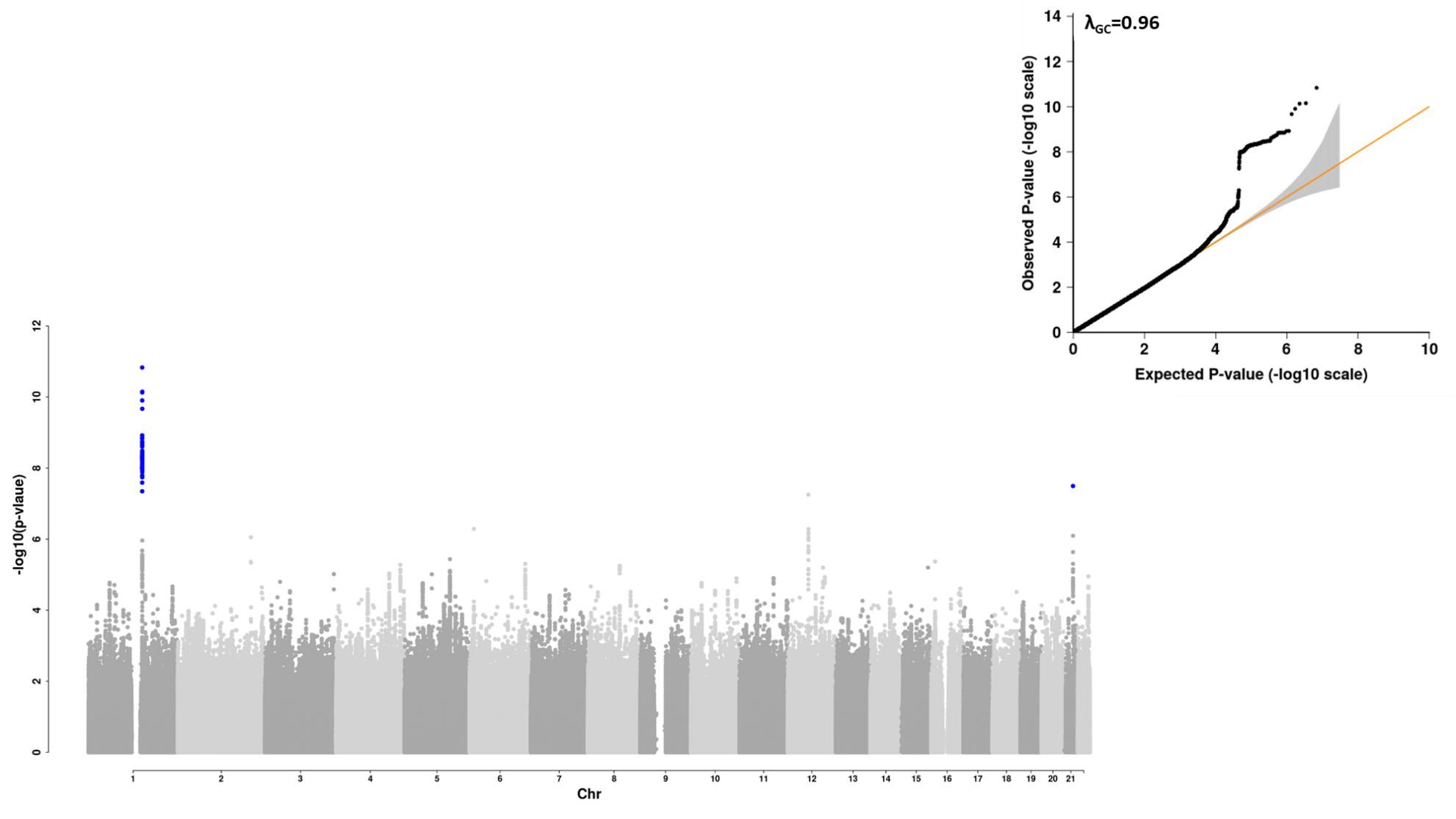
**

**Supplementary Figure 4. GTEx tissue expression data for genes prioritized by the colocalization analysis of the main SCAD GWAS meta-analysis.** Expression quantitative trait loci (eQTL) genes of the SCAD-associated loci and their expression differences across different tissues (n=913).


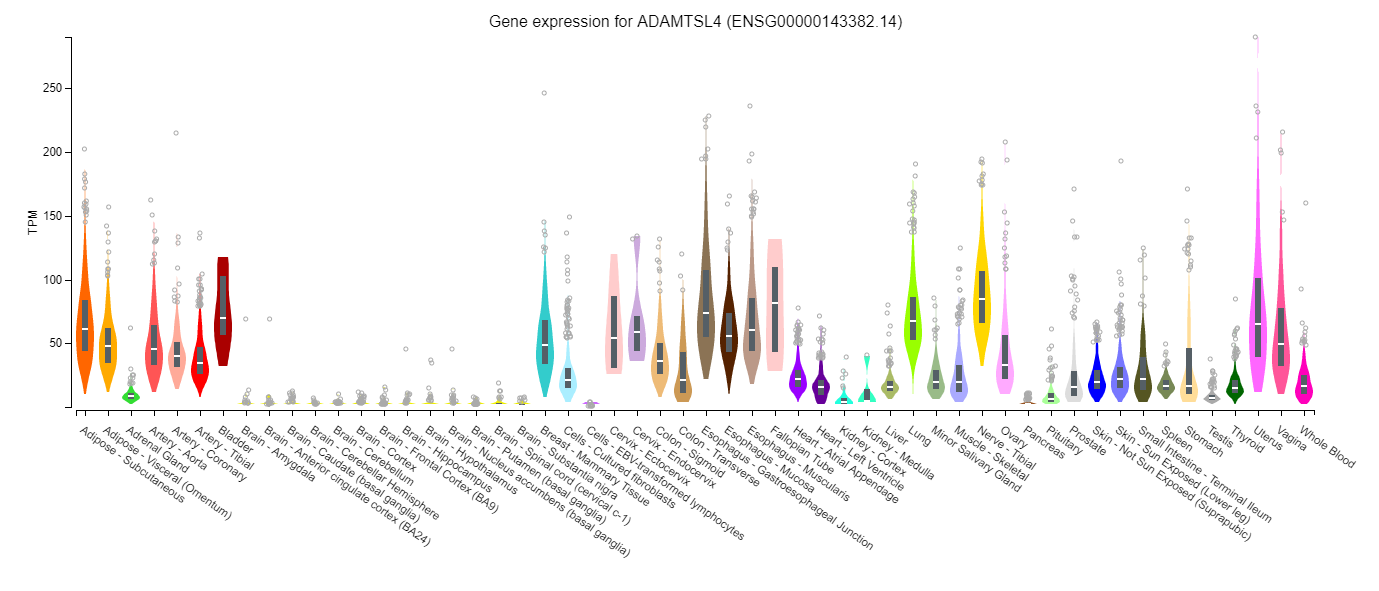


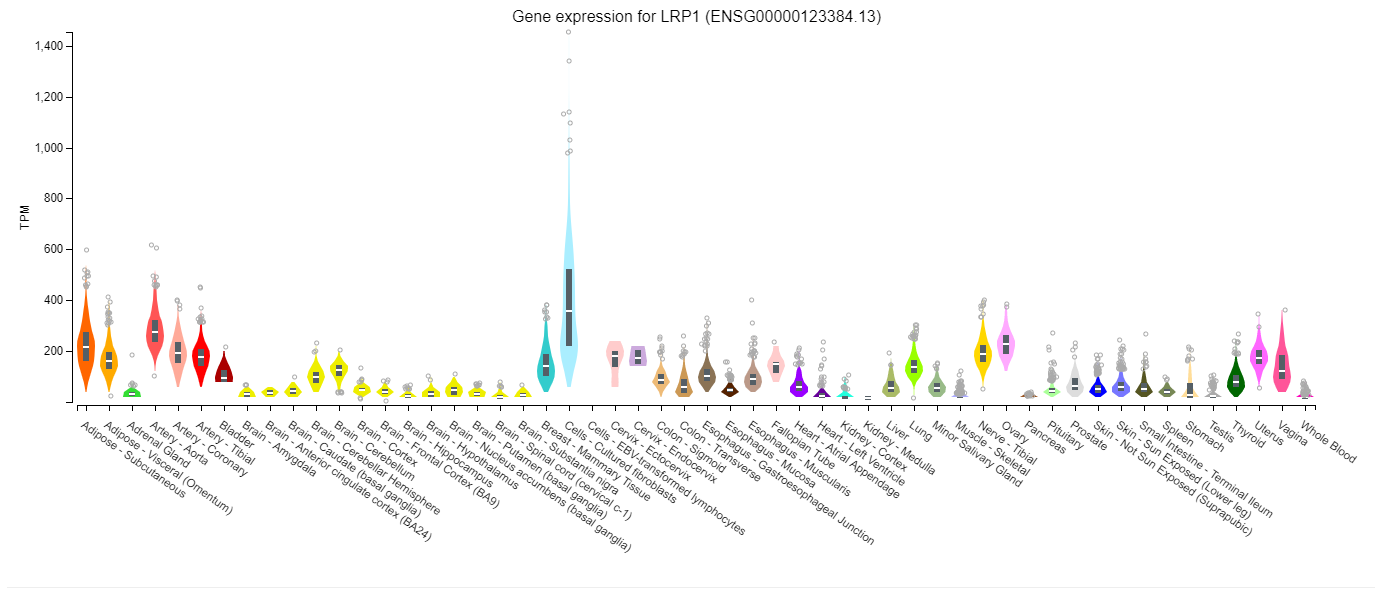


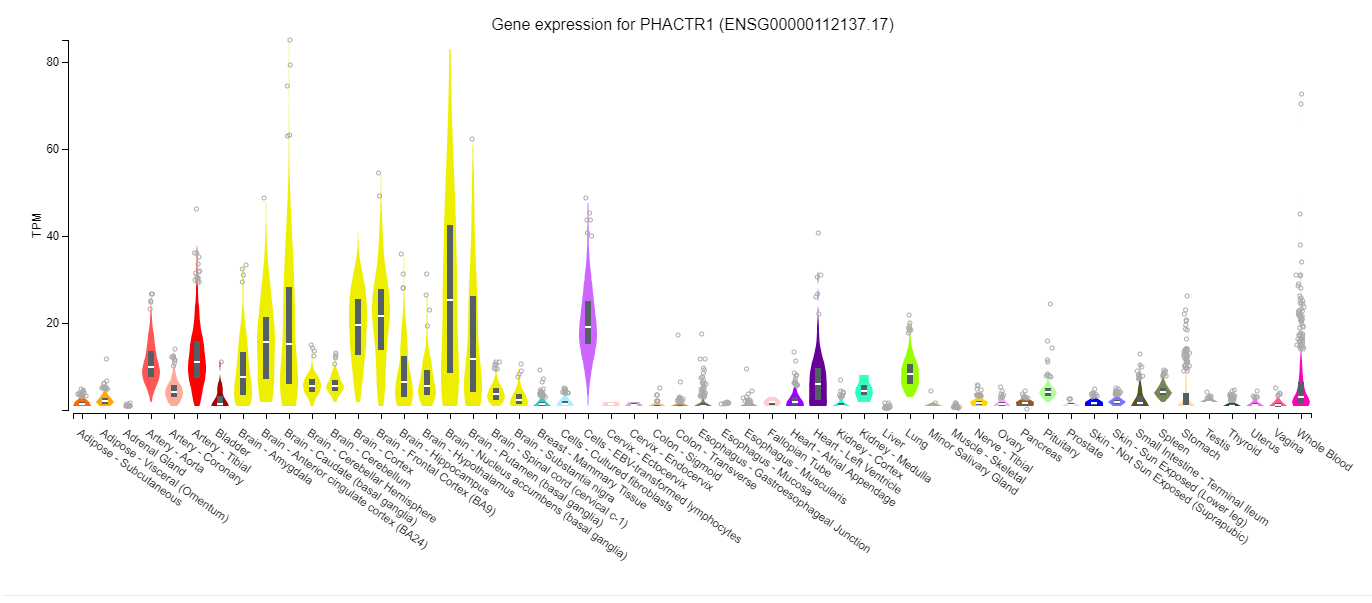


**Supplementary Figure 5. GTEx tissue expression data for genes prioritized by the colocalization analysis of the chromosome 21q22.11 locus identified by the females only SCAD GWAS meta-analysis.** Expression quantitative trait loci (eQTL) genes of the SCAD-associated locus and expression differences across different tissues (n=913).


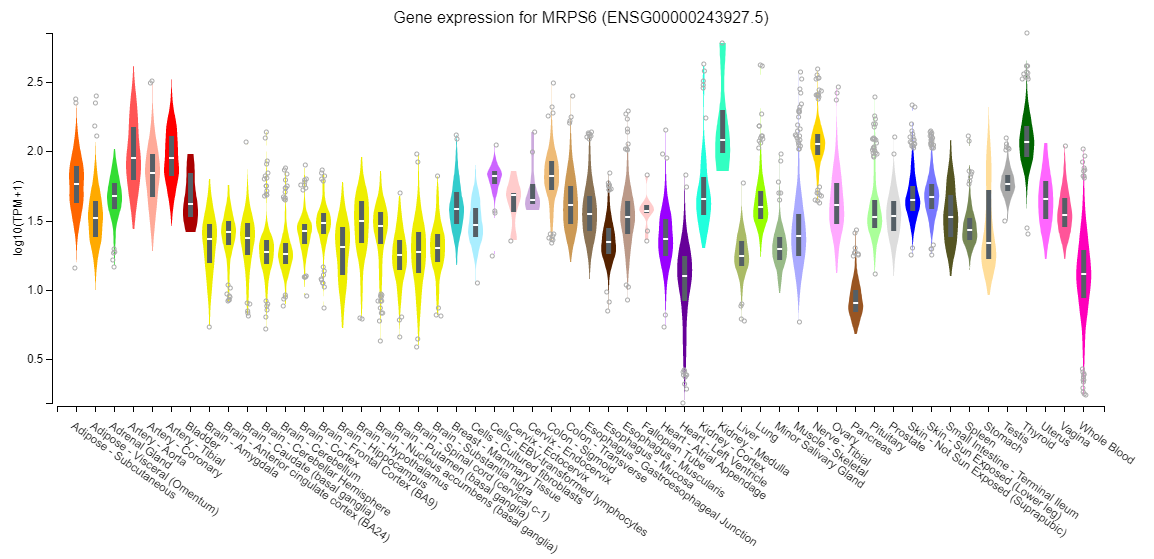


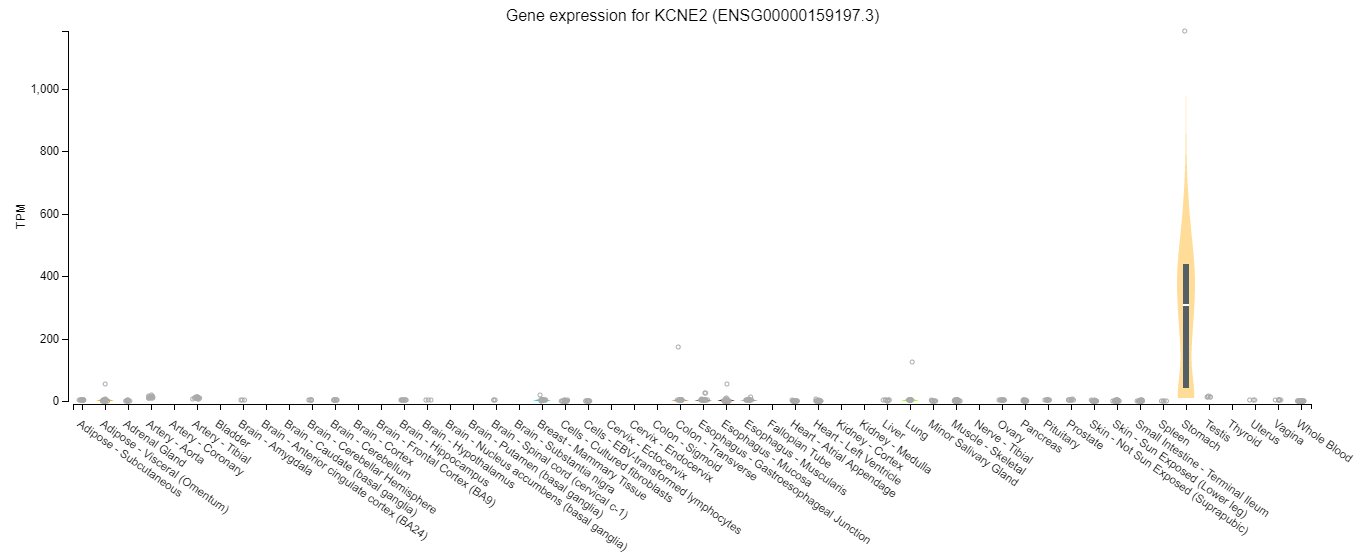


**Supplementary Figure 6. Phenome-wide association study (PheWAS) of UKB data.** PheWAS of weighted PRS_SCAD_ versus self-reported cancer illnesses, non-cancer medical illnesses, medication use, and operations in the UK Biobank at study enrollment (n= 373,015). Two-sided *P* values obtained from logistic regression analyses adjusted for age at enrollment, genetic sex, genotyping array and batch, and the first four principal components of genetic ancestry were considered significant when below a Bonferroni-adjusted threshold (0.05/2,356 ≈ 2.12x10^-5^, dotted blue line).


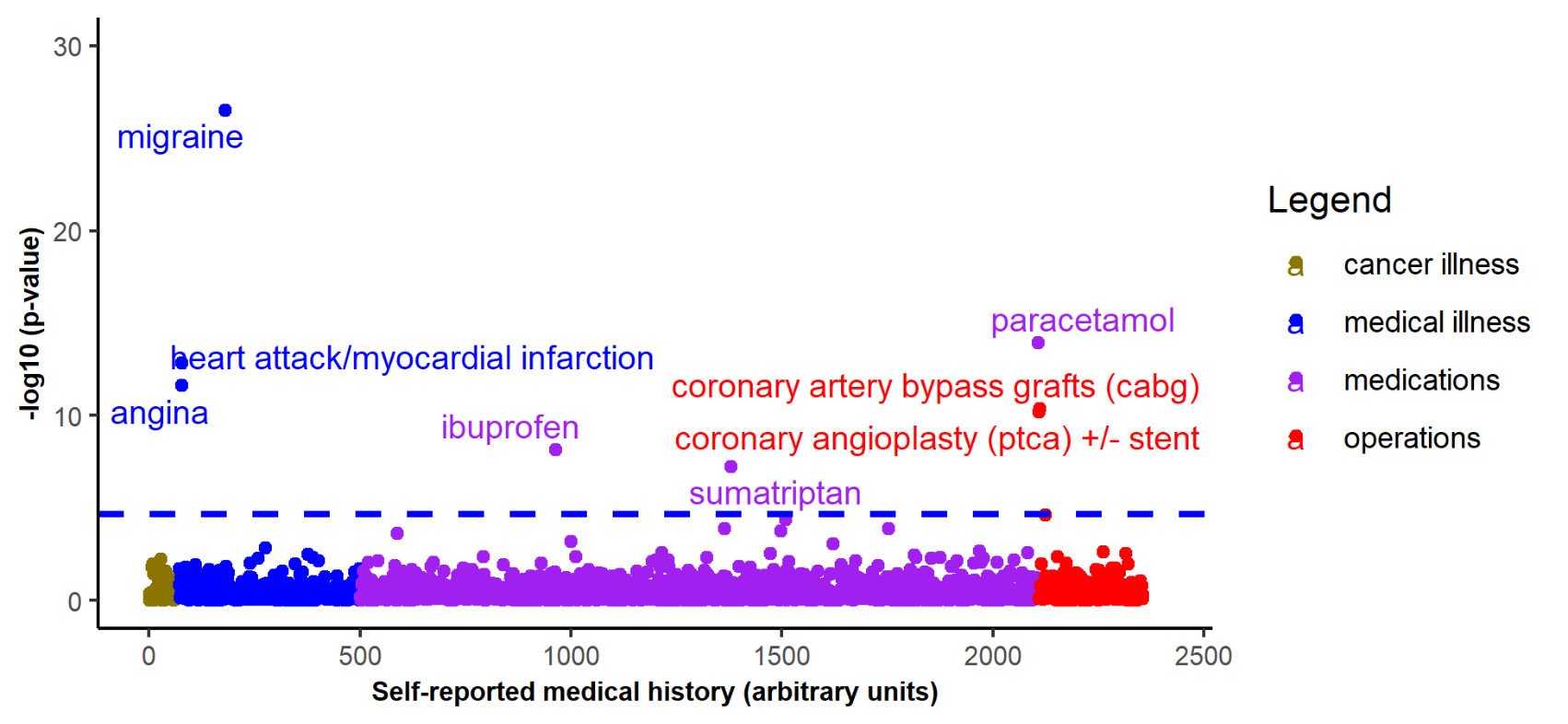


**Supplementary Note – Full list of MVP members:** J. Michael Gaziano^13^, Sumitra Muralidhar^15^, Rachel Ramoni^15^, Jean Beckham^16^, Kyong-Mi Chang^17^, Christopher J. O’Donnell^13,14^, Philip S. Tsao^5^, James Breeling^15^, Grant Huang^15^, JP Casas Romero^13^, Sumitra Muralidhar^15^, Jennifer Moser^15^, Stacey B. Whitbourne^13^, Jessica V. Brewer^13^, Mihaela Aslan^18^, Todd Connor^19^, Dean P. Argyres^19^, Brady Stephens^20^, Mary T. Brophy^13^, Donald E. Humphries^13^, Luis E. Selva^13^, Nhan Do^13^, Shahpoor Shayan^13^, Kelly Cho^13^, Saiju Pyarajan^13^, Elizabeth Hauser^16^, Yan Sun^21^, Hongyu Zhao^18^, Peter Wilson^21^, Rachel McArdle^22^, Louis Dellitalia^23^, Kristin Mattocks^24^, John Harley^25^, Jeffrey Whittle^26^, Frank Jacono^27^, John Wells.^28^, Salvador Gutierrez^29^, Gretchen Gibson^30^, Kimberly Hammer^31^, Laurence Kaminsky^32^, Gerardo Villareal^19^, Scott Kinlay^13^, Junzhe Xu^33^, Mark Hamner^34^, Roy Mathew^35^, Sujata Bhushan^36^, Pran Iruvanti^37^, Michael Godschalk^38^, Zuhair Ballas^39^, Douglas Ivins^40^, Stephen Mastorides^41^, Jonathan Moorman^42^, Saib Gappy^43^, Jon Klein^44^, Nora Ratcliffe^45^, Hermes Florez^46^, Olaoluwa Okusaga^47^, Maureen Murdoch^48^, Peruvemba Sriram^49^, Shing Shing Yeh^50^, Neeraj Tandon^51^, Darshana Jhala^17^, Samuel Aguayo^52^, David Cohen^53^, Satish Sharma^54^, Suthat Liangpunsakul^55^, Kris Ann Oursler^56^, Mary Whooley^57^, Sunil Ahuja^58^, Joseph Constans^59^, Paul Meyer^60^, Jennifer Greco^61^, Michael Rauchman^62^, Richard Servatius^63^, Melinda Gaddy^64^, Agnes Wallbom^65^, Timothy Morgan^66^, Todd Stapley^67^, Scott Sherman^68^, George Ross^69^, Patrick Strollo^70^, Edward Boyko^71^, Laurence Meyer^72^, Samir Gupta^73^, Mostaqul Huq^74^, Joseph Fayad^75^, Adriana Hung^76^, Jack Lichy^77^, Robin Hurley^78^, Brooks Robey^79^, Robert Striker^80^

^5^VA Palo Alto Health Care System, Palo Alto, CA, USA; ^13^VA Boston Healthcare System, Boston, MA; ^15^ US Department of Veterans Affairs, Washington, DC, USA; ^16^ Durham VA Medical Center, Durham, NC, USA; ^17^ Philadelphia VA Medical Center, Philadelphia, PA, USA; ^18^ West Haven VA Medical Center, West Haven, CT, USA; ^19^ New Mexico VA Health Care System, Albuquerque, NM, USA; ^20^ Canandaigua VA Medical Center, Canandaigua, NY, USA; ^21^ Atlanta VA Medical Center, Decatur, GA, USA; ^22^ Bay Pines VA Healthcare System, Bay Pines, FL, USA; ^23^ Birmingham VA Medical Center Birmingham, AL, USA; ^24^ Central Western Massachusetts Healthcare, Leeds, MA, USA; ^25^ Cincinnati VA Medical Center, Cincinnati, OH, USA; ^26^ Clement J. Zablocki VA Medical Center, Milwaukee, WI, USA; ^27^ VA Northeast Ohio Healthcare System, Cleveland, OH, USA; ^28^ Edith Nourse Rogers Memorial Veterans Hospital, Bedford, MA, USA; ^29^ Edward Hines, Jr. VA Medical Center, Hines, IL, USA; ^30^ Veterans Health Care System of the Ozarks, Fayetteville, AR, USA; ^31^ Fargo VA Health Care System, Fargo, ND, USA; ^32^ VA Health Care Upstate New York, Albany, NY, USA; ^33^ VA Western New York Healthcare System, Buffalo, NY, USA; ^34^ Ralph H. Johnson VA Medical Center, Mental Health Research, Charleston, SC, USA; ^35^ Columbia VA Health Care System, Columbia, SC, USA; ^36^ VA North Texas Health Care System, Dallas, TX, USA; ^37^ Hampton VA Medical Center, Hampton, VA, USA; ^38^ Richmond VA Medical Center, Richmond, VA, USA; ^39^ Iowa City VA Health Care System, Iowa City, IA, USA; ^40^ Eastern Oklahoma VA Health Care System, Muskogee, OK, USA; ^41^ James A. Haley Veterans’ Hospital, Tampa, FL, USA; ^42^ James H. Quillen VA Medical Center, Mountain Home, TN, USA; ^43^ John D. Dingell VA Medical Center, Detroit, MI, USA; ^44^ Louisville VA Medical Center, Louisville, KY, USA; ^45^ Manchester VA Medical Center, Manchester, NH, USA; ^46^ Miami VA Health Care System, 11 GRC, Miami FL, USA; ^47^ Michael E. DeBakey VA Medical Center, Houston, TX, USA; ^48^ Minneapolis VA Health Care System, Minneapolis, MN, USA; ^49^ N. FL/S. GA Veterans Health System, Gainesville, FL, USA; ^50^ Northport VA Medical Center, Northport, NY, USA; ^51^ Overton Brooks VA Medical Center, Shreveport, LA, USA; ^52^ Phoenix VA Health Care System, Phoenix, AZ, USA; ^53^ Portland VA Medical Center, Portland, OR, USA; ^54^ Providence VA Medical Center, Providence, RI, USA; ^55^ Richard Roudebush VA Medical Center, Indianapolis, IN, USA; ^56^ Salem VA Medical Center, Salem, VA, USA; ^57^ San Francisco VA Health Care System, San Francisco, CA, USA; ^58^ South Texas Veterans Health Care System, San Antonio, TX, USA; ^59^ Southeast Louisiana Veterans Health Care System, New Orleans, LA, USA; ^60^ Southern Arizona VA Health Care System, Tucson, AZ, USA; ^61^ Sioux Falls VA Health Care System, Sioux Falls, SD, USA; ^62^ St. Louis VA Health Care System, St. Louis, MO, USA; ^63^ Syracuse VA Medical Center, Syracuse, NY, USA; ^64^ VA Eastern Kansas Health Care System, Leavenworth, KS, USA; ^65^ VA Greater Los Angeles Health Care System, Los Angeles, CA, USA; ^66^ VA Long Beach Healthcare System, Long Beach, CA, USA; ^67^ VA Maine Healthcare System,1 VA Center, Augusta, ME, USA; ^68^ VA New York Harbor Healthcare System, New York, NY, USA; ^69^ VA Pacific Islands Health Care System, Honolulu, HI, USA; ^70^ VA Pittsburgh Health Care System, University Drive, Pittsburgh, PA, USA; ^71^ VA Puget Sound Health Care System, Seattle, WA, USA; ^72^ VA Salt Lake City Health Care System, Salt Lake City, UT, USA; ^73^ VA San Diego Healthcare System, San Diego, CA, USA; ^74^ VA Sierra Nevada Health Care System, Reno, NV, USA; ^75^ VA Southern Nevada Healthcare System, North Las Vegas, NV, USA; ^76^ VA Tennessee Valley Healthcare System, South Nashville, TN, USA; ^77^ Washington DC VA Medical Center, Washington, D. C., USA; ^78^ W.G. (Bill) Hefner VA Medical Center, Salisbury, NC, USA; ^79^ White River Junction VA Medical Center, White River Junction, VT, USA; ^80^ William S. Middleton Memorial Veterans Hospital, Madison, WI, USA
