# Supplementary material for: Chromosome 1q21.2 and additional loci influence risk of spontaneous coronary artery dissection and myocardial infarction
Source: Nat Commun. 2020 Sep 4;11:4432. doi: 10.1038/s41467-020-17558-x (PMC7474092; doi:10.1038/s41467-020-17558-x)
Supplement: Supplementary file 2 — Description of Additional Supplementary Data [file 41467_2020_17558_MOESM2_ESM.docx]

**Description of Additional Supplementary Files**

**File name:** Supplementary_Data1.xlsx

**Description:** Supplementary data 1. GWAS meta-analysis associations with P<5x10-8 (Full table).

**File name:** Supplementary_Data2.xlsx

**Description:** Supplementary data 2. SNPs with association P < 0.0001 in the SCAD GWAS meta-analysis.

**File name:** Supplementary_Data3.xlsx

**Description:** Supplementary data 3. RNA expression related to the GWAS-identified SNPs.

**File name:** Supplementary_Data4.xlsx

**Description:** Supplementary data 4. For the 7 independent loci comprising the PRS_SCAD_, a comparison of results between the SCAD GWAS, CAD association in MVP cohort, UKB Cox proportional hazards regression models for MI, and logistic regression on SCAD versus non-SCAD in the FMD cohort.

**File name:** Supplementary_Data5.xlsx

**Description:** Supplementary data 5. PheWAS in the UKB database for top ranked SNPs identified in the main SCAD GWAS meta-analysis.

**File name:** Supplementary_Data6.xlsx

**Description:** Supplementary data 6. Individual locus results for CAD loci (386 independent SNPs) in the SCAD GWAS meta-analysis results.

**File name:** Supplementary_Data7.xlsx

**Description:** Supplementary data 7. Phenome-wide association study (PheWAS) of PRS_SCAD_ by UKB data.

**File name:** Supplementary_Data8.xlsx

**Description:** Supplementary data 8. ICD codes used to exclude arterial diseases and connective tissue disorders from the MGI control group.
