# Supplementary material for: Chromosome 1q21.2 and additional loci influence risk of spontaneous coronary artery dissection and myocardial infarction
Source: Nat Commun. 2020 Sep 4;11:4432. doi: 10.1038/s41467-020-17558-x (PMC7474092; doi:10.1038/s41467-020-17558-x)
Supplement: Supplementary file 11 — Reporting Summary [file 41467_2020_17558_MOESM11_ESM.pdf]

Corresponding author(s): NCOMMS-19-42204-T (Santhi K. Ganesh, Jacqueline Saw)

Last updated by author(s): 2020/05/14

Reporting Summary

Nature Research wishes to improve the reproducibility of the work that we publish. This form provides structure for consistency and transparency in reporting. For further information on Nature Research policies, see Authors & Referees and the Editorial Policy Checklist.

Statistics

For all statistical analyses, confirm that the following items are present in the figure legend, table legend, main text, or Methods section.

n/a	Confirmed	
☐	☒	The exact sample size (n) for each experimental group/condition, given as a discrete number and unit of measurement
☐	☒	A statement on whether measurements were taken from distinct samples or whether the same sample was measured repeatedly
☐	☒	The statistical test(s) used AND whether they are one- or two-sided<br>*Only common tests should be described solely by name; describe more complex techniques in the Methods section.*
☐	☒	A description of all covariates tested
☐	☒	A description of any assumptions or corrections, such as tests of normality and adjustment for multiple comparisons
☐	☒	A full description of the statistical parameters including central tendency (e.g. means) or other basic estimates (e.g. regression coefficient) AND variation (e.g. standard deviation) or associated estimates of uncertainty (e.g. confidence intervals)
☐	☒	For null hypothesis testing, the test statistic (e.g. F, t, r) with confidence intervals, effect sizes, degrees of freedom and P value noted<br>*Give P values as exact values whenever suitable.*
☒	☐	For Bayesian analysis, information on the choice of priors and Markov chain Monte Carlo settings
☒	☐	For hierarchical and complex designs, identification of the appropriate level for tests and full reporting of outcomes
☒	☐	Estimates of effect sizes (e.g. Cohen's d, Pearson's r), indicating how they were calculated

Our web collection on statistics for biologists contains articles on many of the points above.

Software and code

Policy information about availability of computer code

Data collection	(1) Genotyping Module v1.9.4 and Illumina GenomeStudio (version 2.0) were used to cluster and call genotypes for CanSCAD, FMD and MGI samples samples. PLINK v1.9 and vcf tools for follow-up data merging and converting.<br><br>(2) Imputation data were obtained by imputing our genotyping data in the Michigan Imputation Server using Minimac4 method; HRC r1.1 2016 reference panel; Eagle v2.3 as phase output; EUR as quality control population.<br><br>(3)Redcap v9.1 (Vanderbilt University) to collect, query and export the UBC clinical data.<br><br>(4)MGI controls, FMD, UKB and MVP samples were collected based on International Classification of Diseases (ICD9/10) diagnostic and Current Procedural Terminology (CPT) codes to identify subjects with interested phenotypes.<br><br>(5)GWAS catalog and PheWAS from UK Biobank database for known SNPs look-up.<br>(6)All GTEx data were queried and downloaded from the GTEx Portal browser (GTEx Analysis V7) for eQTL and RNAseq data.
Data analysis	(1) R version 3.6.2 for general statistical computing (ex. linear regression, logistic regression, clinical data summary statistics, Odds ratio and confidence interval computing, polygenic risk score analysis,...,etc.), figure generation (ex. manhattan plot, QQ plot, Odds ratio forest plot), and case-control matching.<br><br>(2) PLINK 1.9 and VCFtools for data merging, basic QC and genotyping data processing.<br>(3) HRC checking tool from McCarthy Group [https://www.well.ox.ac.uk/~wrayner/tools/] for pre-imputation QC check.<br>(4) TRACE in LASER server v3.0.0 for PCA clustering and PCs computing for genome-wide genotyping data.<br>(5) Michigan Imputation Server (Minimac4) for imputation by HRC reference.<br>(6) SAIGE 0.35.8.3 for case-control GWAS (both discovery and replication) analysis and conditional analyses.<br>(7) PLINK 1.9 was used for IBD analysis and our independent FMD cohort replication for the top1 locus using logistic regression model.

(8) METAL for meta-analysis of GWAS summary statistics.
(9) LD pruning was done by PLINK 1.9 'clumping' method to identify the top independent loci.
(10) FDR analysis was done by R package 'qvalue' using meta-analysis result (p-values) of 324,087 genome-wide LD-pruning SNPs that have MAF>1% and after clumping of r2>=0.2 at +/- 500Kb with no p-value filter.

(11) LocusZoom for generating regional plots with gene annotation.
(12) LDlink program for LD r-square check between top SNPs.
(13) Time-to-event analyses were performed with the "survival" version 2.43-3 package for R version 3.5.1.
(14) PHESANT software for R version 3.5.1 to perform a phenome-wide association study of weighted and continuous PRS-SCAD with 2,352 phenotypes related to self-reported history of cancers, non-cancer illnesses, operations, and medications assessed at study enrollment (https://github.com/MRCIEU/PHESANT). Tests for interaction were assessed between genetic features and sex.

(15) GTX tool package (https://cran.r-project.org/web/packages/gtx/index.html) in R to perform PRS-CAD analysis based on summary statistics from SCAD meta-analysis data.

(16) GTEx data analyses were performed in R Studio (3.5.0). Sex differences in arterial tissue were analyzed via DESeq2 (1.22.2). Gene expression plots were generated using ggplot2 (3.2.1).

(17) The OR forest plot for various PRS analyses were generated using R package 'forestplot'.
(18) The co-localization analysis was done by two tools: (i) R package 'coloc' using coloc.abf() function to test the posterior probabilities for the hypothesis of both traits are associated and share a single causal variant, and (ii) locuscompareR to make a visualized figure to compare between eQTL result and GWAS result.

(19) For heritability estimation, we fit a univariate linear mixed model (LMM) for estimating the proportion of variance explained (PVE) by typed genotypes using GEMMA, a software implementing the Genome-wide Efficient Mixed Model Association algorithm for genome-wide association studies.

(20) Slides for In situ hybridization for mRNA were imaged on Nikon Ti microscope using Element Software (Nikon, Melville NY).

For manuscripts utilizing custom algorithms or software that are central to the research but not yet described in published literature, software must be made available to editors/reviewers. We strongly encourage code deposition in a community repository (e.g. GitHub). See the Nature Research guidelines for submitting code & software for further information.

Data

Policy information about availability of data

All manuscripts must include a data availability statement. This statement should provide the following information, where applicable:
- Accession codes, unique identifiers, or web links for publicly available datasets
- A list of figures that have associated raw data
- A description of any restrictions on data availability

GWAS summary statistics will be made available via NCBI dbGaP (accession number: TBD), The analysis results of expression QTLs is available in our Supplementary Tables and the corresponding GTEx data is available on the GTEx project portal (https://www.gtexportal.org/home/datasets).

Field-specific reporting

Please select the one below that is the best fit for your research. If you are not sure, read the appropriate sections before making your selection.

☒ Life sciences ☐ Behavioural & social sciences ☐ Ecological, evolutionary & environmental sciences

For a reference copy of the document with all sections, see nature.com/documents/nr-reporting-summary-flat.pdf

Life sciences study design

All studies must disclose on these points even when the disclosure is negative.

Sample size

(1) Overall study design: We performed a SCAD phenotype case-control association study of human subjects (CanSCAD cases versus MGI controls) using genome-wide imputation genotyping data. Common variants with MAF>1% and imputation Rsq>=0.8 were analyzed (N=6,690,240 SNPs). The case samples have the same genotyping array platform with the control sample pool, and they are merged to do the imputation together.
(2) Before study, power calculation for different sample sizes: Using Baye's theorem, we calculated the expected proportions of genotypes in cases and controls respectively for different disease models assumed (via setting different parameters: disease prevalence rate, MAF, Odds ratio, type I error rate, sample size, genetic model).The power of a case-control association can be calculated analytically by integrating the non-central chi-square distribution from NCP of the Pearson chi-square test, which compares the frequency of risk genotypes in cases and controls (Power is defined by given the model exists, the probability that we can reject the null hypothesis). We used R package 'pwr' in the construction of the power calculation formula and Michigan GAS Power Calculator (http://csg.sph.umich.edu/abecasis/gas_power_calculator/index.html) to estimate the minimal sample size required for getting enough power (given that to achieve P<5e-8 in GWAS).
Considering that we aimed to study a rare disease with limited numbers of cases, we maximized statistical power by enlarging our control sample size and utilized the SAIGE program that accounts for imbalanced study design.
(3)Case control matched GWAS study design: To reduce the possible false-positives in our GWAS result, we required controls to have the same gender, close birth years and close ancestries. Based on our healthy control pools, using a greedy matching strategy, we matched up to 21 controls for each case. After basic sample QC and identifying two genetic cases that will be removed in our GWAS study, we had ultimately

270 SCAD cases and 5,263 matched MGI controls in the discovery analysis (N=5533), and 163 SCAD cases and 3207 matched MGI control in the replication analysis(N=3370). Meta-analysis was performed for the SCAD GWAS discovery and replication results. Secondary analyses were done for female-only SCAD GWAS (N=4895 in discovery and N=2996 in replication), male-only SCAD GWAS (N=638 in discovery GWAS), SCAD +FMD subgroup GWAS (N=2922 in discovery), and SCAD-FMD subgroup GWAS (N=2655 in discovery). A sensitivity analysis was also done using our SCAD GWAS samples after removing Asian ancestry (N=5076 in discovery).

(4) For the top locus, we had performed an independent replication using FMD cohort unrelated 28 SCAD cases versus 355 Cleveland Clinic Genebank controls (N=383).
(5) The heritability estimate was done from the SCAD GWAS discovery stage samples (5,577 samples and 5,020,100 SNPs).
(6) Evaluations of the PRS-SCAD were performed in (i) cohort of 412 individuals with FMD (N=28 SCAD cases) for PRS-SCAD to SCAD status in FMD cases, (ii) 373,056 UK biobank samples (N=15,476 CAD/MI cases) for PRS-SCAD to MI status, (iii) 294,465 MVP samples (N=95,347 CAD cases) for PRS-SCAD to CAD status, and 314,434 MVP samples (N=14,802 MI cases) for PRS-SCAD to MI status. (iv) The PheWAS samples size based on UK biobank samples was 373,056.
(7) Evaluation of the PRS-CAD was performed in our SCAD meta-analysis (N=8903).
(8) For the eQTL data and colocalization analysis, the sample size is 913 subjects (593 males, 320 females) for eQTL data, and 8,903 for GWAS data.
(9) For in situ immunization assay, the sample size is one.

Data exclusions

(1) In our GWAS control group from MGI, several ICD codes corresponding to diagnoses of arterial aneurysm, dissection and non-atherosclerotic dysplasia and stenosis were excluded, as well as connective tissue disorders.
(2) Two genetic cases were further identified by reviewing all clinical and genetic records of SCAD cases, and annotated all all low frequency variants on our gwas GWAS +exome chip which was enriched for coding variants in genes known to underlie genetically mediated arteriopathy and aortopathy.

Replication

(1) For GWAS, replication study was done for 163 CanSCAD v.s. 3207 matched MGI controls, using the same study design and analysis method. Independent loci (after LD pruning of r>=0.2, +/- 500 Kb) with P<5e-8 in discovery study were inspected in replication results. We examined the p-value in the replication study for the only one locus we identified in our discovery stage result (P=2.88E-10). The top locus in chr1 region was replicable in replication results (P=7.32E-4) and passed the Bonferroni correction p-value threshold (P=0.05/1).
(2) Meta-analysis was performed for the SCAD GWAS discovery and replication results. All loci meets P<5e-8 in meta-analysis or the secondary female-only meta-analysis were reported.
(3) For the top locus, we had performed an independent replication using FMD cohort unrelated 28 SCAD cases versus 355 Cleveland Clinic Genebank controls (N=383). The top chr1 locus was also replicated (P=0.032) and passed the Bonferroni correction p-value threshold.
(4) The PRS analysis of our top SCAD SNPs (P<5e-6 in SCAD GWAS meta-analysis, of which the threshold was selected by false discovery rate q-value<0.05 from SCAD GWAS meta-analysis result of 324,087 independent genome-wide LD-pruning SNPs that have MAF>1% and after clumping of r2>=0.2 at +/- 500Kb with no p-value filter) also supported that higher SCAD polygenic risk scores were associated with higher risk of SCAD in FMD case group (P=0.02).
(5) Sensitivity and secondary analyses were conducted for female-only SCAD GWAS, male-only SCAD GWAS, SCAD+FMD GWAS, SCAD-FMD GWAS, and SCAD GWAS after removing Asian ancestries.
(6) Other follow-up analysis from UK biobank survival analysis of MI, PRS-SCAD for MI/CAD in UKBB and MVP cohort, Phewas, gtx eQTL RNAseq data queried and colocalization analysis were included as supporting materials for our top finding. For PRS-SCAD analyses, sensitivity analysis was also conducted in PRS-SCAD that removing PHACTR1 SNP (6 loci remained).
(7) Individual locus results for known CAD loci (386 SNPs) in our SCAD GWAS meta-analysis results were also retrieved as well as PRS-CAD in our SCAD GWAS data.

Randomization

(1) In our case control matching design, we required controls to have the same gender, close birth years and close ancestries. Also in our GWAS model, we adjusted genotyping PCs.
(2) For PRS-SCAD analysis, we adjusted age, sex in FMD cohort and adjusted age,sex, PCs in UKBB cohort and MVP cohort.
(3) We adjusted age, sex, genotyping PCs in our UK biobank Cox regression analysis and PheWAS analysis.

Blinding

As this is an association study, blinding may not be applicable. However, in our sample handling and data analysis steps, all of the samples were de-indentified. Laboratory technicians performing genotyping were blinded to sample information.

Reporting for specific materials, systems and methods

We require information from authors about some types of materials, experimental systems and methods used in many studies. Here, indicate whether each material, system or method listed is relevant to your study. If you are not sure if a list item applies to your research, read the appropriate section before selecting a response.

Materials & experimental systems

n/a	Involved in the study	
☐	☒	Antibodies
☒	☐	Eukaryotic cell lines
☒	☐	Palaeontology
☒	☐	Animals and other organisms
☐	☒	Human research participants
☐	☒	Clinical data

Methods

n/a	Involved in the study	
☒	☐	ChIP-seq
☒	☐	Flow cytometry
☒	☐	MRI-based neuroimaging

Antibodies

Antibodies used	anti-ADAMTSL4, Sigma, Catalog# HPA006279, Lot#A78645
Validation	HPA006279 is an antibody used for the Human Protein Atlas. Under the Protein Atlas guidelines, this antibody has an approved rating for antibody validation in 44 tissues. Experimental staining without primary Ab on control tissue slides indicated no staining where with primary indicated positive staining.<br>Gabriel LA, Wang LW, Bader H, et al. ADAMTSL4, a secreted glycoprotein widely distributed in the eye, binds fibrillin-1 microfibrils and accelerates microfibril biogenesis. Invest Ophthalmol Vis Sci. 2012;53(1):461–469. Published 2012 Jan 31. doi:10.1167/iovs.10-5955

Human research participants

Policy information about studies involving human research participants

Population characteristics

Discovery stage SCAD cases: age= 53.28 yrs (SD=9.68), 89 % female.
Discovery stage MGI controls: age= 52.86 yrs (SD=15.31), 88 % female.
From our discovery stage SCAD cases, 60% has FMD.
Replication stage SCAD cases: age= 50.52 yrs (SD=10.44), 90 % female.
Replication stage MGI controls: age= 49.20 yrs (SD=15.49), 89 % female.
SCAD cases in FMD cohort for independent SCAD replication: age= 49.64 yrs (SD=7.92), 100 % female.
Cleveland Clinic Genebank controls: age= 54.59 yrs (SD=10.12), 96.9 % female.

Overall FMD cohort for PRS-SCAD analysis: age= 54.59 yrs (SD=10.12), 96.9 % female, 7% has SCAD.
91% of our SCAD cases and 98% of our FMD cases are from European ancestry.
For detailed population characteristics for SCAD, please see Table S1.
PheWAS: mean age at enrollment=56.91(SD=8.00), 54.14 % female.
Overall UK Biobank:mean age=56.53 yrs (SD=8.10), 54.40 % female, 85% British White.
Overall MVP population: age=63.5 (SD=13.48), 2% female, 69.2% European American (white)

Recruitment

Procedures:

(1)SCAD cases: The current study is a prospective genetic study evaluating the genetics of SCAD in the large prospective cohort of patients enrolled in the Canadian SCAD (CanSCAD) Study. The CanSCAD study included SCAD patients from the prospective Canadian SCAD Cohort Study (funded by the Canadian Institutes of Health Research) and the Non-Atherosclerotic Coronary Artery Disease (NACAD) Study. Patients presenting with acute SCAD were prospectively enrolled from 22 sites throughout North America (20 sites in Canada and 2 in the United States). SCAD diagnosis was confirmed on coronary angiography by the UBC core laboratory research team, and categorized according to previously established Saw classification. Type 1 SCAD depicts contrast dye staining of arterial wall with multiple radiolucent lumen, with or without dye hang-up or slow contrast clearing from the lumen. Type 2 SCAD depicts diffuse and smooth narrowing that varies in severity; Type 2A describes presence of normal arterial segments proximal and distal to dissection; Type 2B describes dissection that extends to distal tip of the artery. Type 3 SCAD depicts focal or tubular stenosis that appears similar to atherosclerosis. Intracoronary imaging with optical coherence tomography or intravascular ultrasound was performed at the discretion of the treating physicians to aid angiographic diagnosis. Detailed baseline demographics, targeted history for predisposing conditions and precipitating stressors, and laboratory screening for predisposing conditions were performed. Screening for FMD was recommended for all SCAD patients, and multifocal FMD was defined according to consensus guidelines24. Patients were prospectively followed post-discharge at 1, 6, and 12 months, and annually thereafter for 3 years for cardiovascular (CV) events. Research ethics board approvals were obtained at each site, and all patients provided informed consent for participation.
In the CanSCAD Genetics Substudy, site-specific research ethics board approvals for the study and individual patient consents were obtained. Genetic studies were performed on CanSCAD patients who provided informed consent. Collection of DNA was obtained through blood or saliva self-collection kit (Oragene-500 kit, DNAGenotek). DNA was extracted according to the manufacturer's instruction (DNAGenotek) as previously described, and quantified using the Quant-iT PicoGreen assay (Life Technologies). DNA samples were normalized to a concentration of 50ng/μl for genotyping. The processed DNA samples were batched and transferred to the University of Michigan for GWAS analysis.

(2) FMD cases: Between 2010 and 2015, unrelated adult subjects with mFMD (N=412) were enrolled in one of two participating repositories with respective IRB approvals at each institution: the University of Michigan Genetic Study of Arterial Dysplasia or the Cleveland Clinic FMD Biorepository. All participants provided informed consent and study activities were approved by the enrolling institution's IRBs. Each research participant contributed either a blood or saliva sample via standard K+ EDTA blood collection tubes or commercial saliva collection kits (Oragene, DNAGenotek).DNA was isolated according to commercial kit protocols (Nucleospin Tissue (TakaraBio)), extracted according to the prepIT-L2P extraction kit (DNAGenotek) and quantified using the Quant-iT PicoGreen dsDNA kit (ThermoFisher).

(3) MGI controls: The Michigan Genomics Initiative (MGI) is a program that recruited participants while awaiting diagnostic, interventional, and surgical procedures. Participants provided a blood sample for genetic analysis and agreed to link their sample to their electronic health record and other sources of health information. The current study's analyses involved 13756 individuals from MGI genotyped with the same version (v1.1) of the Illumina BeadArray genotyping platform as the SCAD and FMD cases at the University of Michigan DNA Sequencing Core Facility. Several ICD codes corresponding to diagnoses of arterial aneurysm, dissection and non-atherosclerotic dysplasia and stenosis were excluded, as well as connective tissue disorders.

(4) Genebank controls: The Cleveland Clinic GeneBank study is a sample repository generated from consecutive patients undergoing elective diagnostic coronary angiography or elective cardiac computed tomographic angiography with extensive clinical and laboratory characterization and longitudinal observation. Subject recruitment occurred between 2001 and 2006. Ethnicity was self-reported and information regarding demographics, medical history, and medication use was obtained by patient interviews and confirmed by chart reviews. All patients selected as controls were age and sex matched to the FMD cohort and had no evidence of coronary artery disease, defined as adjudicated diagnoses of stable or unstable angina, myocardial infarction (adjudicated definition based on defined electrocardiographic changes or elevated cardiac enzymes), angiographic evidence of 50% stenosis of one or more major epicardial vessel, and/or a history of known coronary artery disease (documented infarction, coronary disease, or history of revascularization). All patients provided written informed consent prior to being enrolled in GeneBank and the study was approved by the Institutional Review Board of the Cleveland Clinic.

(5) UK biobank samples:
The UK Biobank recruited adults of 40-69 years-of-age from across the United Kingdom between 2006-201052. Participants were assessed at study at study enrolment via medical histories, physical exams, and biochemical measurements. Participant data is linked to hospital episode statistics that span from before study enrollment to March 31, 2017.
For survival analysis, MI events were defined pre-enrollment by self-reported medical history and post-enrollment by hospital episode statistics using International Classification of Diseases, Version 10 diagnosis codes (I21, I22, I23, or I24). Events were censored on the date of loss-to-follow-up, death, or if individuals remained event-free up to March 31, 2017.

(6) MVP cohort:The design of the Million Veteran Program (MVP) has been previously described56. Briefly, active users of the Veteran Health Administration (VA) of any age have been recruited from more than 60 VA Medical Centers nationwide since 2011 with current enrollment at >825,000. Informed consent is obtained from all participants to provide blood for genomic analysis and access to their full electronic health record (EHR) data within the VA prior to and after enrollment. Imputed genetic information is available for up to 314,434 participants assigned to white-European ancestry using the HARE algorithm57,58. We used inpatient and outpatient International Classification of Diseases (ICD9/10) diagnostic and Current Procedural Terminology (CPT) codes to identify subjects with clinical CAD either before enrollment going back to 2002 or after enrollment until mid-August 2018. An individual was classified as a case if he or she had ≥1 admission to a VA hospital with discharge diagnosis of acute myocardial infarction (AMI) OR ≥1 procedure code for revascularization of the coronary arteries OR ≥ 2 ICD codes for CAD (410 to 414) on ≥ 2 dates. Individuals with only 1 ICD code for CAD on 1 date and no discharge diagnoses for AMI or revascularization procedures were excluded from the analyses and remaining subjects were classified as controls. This algorithm identified up to 95,347 unrelated subjects with CAD and 199,118 unrelated controls with 19,969 subjects being excluded due to ambiguous CAD status. For MI status, wWe performed subgroup analysis involving the subset of cases with evidence of a hospitalization for MI. These cases were compared to all controls in our association analysis (N=14,802 unrelated MI cases and N=299,632 unrelated controls).

Selection:

(1) Patients with SCAD presenting to multiple institutions participating in the Can-SCAD registry were enrolled consecutively, without selections. There is less than 2% decline rate for the Canadian SCAD Study and therefore no selection bias.

(2) UBC SCAD Genetic sub-study: The sample collection procedures are still ongoing and rate of decline is similar or below the rate that was previous reported in other genetic studies*.

*Why Patients Decline Genomic Sequencing Studies: Experiences from the CSER Consortium
Laura M. Amendola, Jill O. Robinson, et.al (2018). J Genet Couns. 2018 Sep; 27(5): 1220–1227.

(3) Patients with FMD were enrolled into the University of Michigan Genetic Study of Arterial Dysplasia through self-referral to the study, or presentation to subspecialty clinics in cardiovascular diseases, and the Cleveland Clinic FMD Biorepository through presentation to a vascular medicine subspecialty clinic. It is unknown whether patients presenting to a subspecialty clinic or by self-referral to a study have any different predisposition to SCAD, but this is possible.

Ethics oversight

University of British Columbia
University of Michigan
Cleveland Clinic
UK Biobank: This study was approved by the UK Biobank (application ID: 42857) and by the Clinical Research Ethics Board of the University of British Columbia (H18-02181).
MVP:The MVP received ethical and study protocol approval from the VA Central Institutional Review Board in accordance with the principles outlined in the Declaration of Helsinki.
List of REBs approved the SCAD subjects study (Canadian Term is REB vs IRB in the US):
University of British Columbia Clinical Research Ethics Board
University of Alberta Health Research Ethics Board
University of Manitoba Biomedical Research Ethics Board
Regina Qu'Applelle Health Region Research Ethics Board
Sunnybrook Health Sciences Center Research Ethics Board
Hamilton Integrated Research Ethics Board
Nova Scotia Health Authority Research Ethics Board
Horizon Health Network Research Ethics Board
Tri-Hospital Research Ethics Board
University of Calgary Health Research Ethics Board
University of Michigan Medical School Institutional Review Board

Note that full information on the approval of the study protocol must also be provided in the manuscript.

Clinical data

Policy information about clinical studies

All manuscripts should comply with the ICMJE guidelines for publication of clinical research and a completed CONSORT checklist must be included with all submissions.

Clinical trial registration	SCAD samples ClinicalTrials.gov Identifier: NCT02188069.
Study protocol	We did not include the genetic sub-study into the Canadian SCAD description.
Data collection	Participants are regularly followed on the phone and asked about any hospital visits. For cases suspected for outcomes, health records and angiogram films were obtained and events adjudicated.
Outcomes	All outcomes are pre-defined in the protocol.
